# Visible light-driven ligand-to-metal charge transfer-mediated selective cleavage of β-O-4 lignin model compounds: a greener route to lignin valorization†

**DOI:** 10.1039/d5gc00948k

**Published:** 2025-03-12

**Authors:** Ayesha Khan, Logan W. Evans, David B. C. Martin

**Affiliations:** a Department of Chemistry, University of Iowa Iowa City Iowa 52242 USA david-martin@uiowa.edu

## Abstract

Lignin is the most abundant renewable source of aromatics in nature. The β-O-4 bond is the most predominant linkage in lignin; therefore, methods for the selective cleavage of the β-O-4 bond are of great importance in order to break down lignin and produce value-added aromatic compounds. Herein, we report a visible light-driven, ligand-to-metal charge transfer (LMCT)-mediated, two-step approach for cleaving C_β_–O bonds in β-O-4 alcohol model compounds using titania (TiO_2_) as a photocatalyst. In the first step, the alcohol forms a visible light-absorbing LMCT complex on the surface of titania, which enables oxidation to the corresponding ketone under green light. The LMCT-mediated oxidation afforded high conversion of β-O-4 alcohol model compounds (79–97%) with high selectivity for β-O-4 ketones (>95%). Our studies reveal that the superoxide radical anion likely plays a key role in the oxidation. In the second step, the LMCT-assisted reductive cleavage of β-O-4 ketone is achieved by employing triethylammonium tetraphenylborate as a visible light sensitizer and proton donor. The LMCT-facilitated reductive cleavage of β-O-4 ketones exhibits high selectivity (up to 100%) for target fragmentation products under blue light. Experiments including EPR analysis suggest that *in situ* formed Ti^3+^ is responsible for the reductive cleavage of β-O-4 ketones. Moreover, a two-step, one-pot cleavage reaction was successfully carried out with good to high selectivity for C_β_–O bond cleavage products with a single catalyst. Our work offers a promising solution for the selective cleavage of β-O-4 bonds under mild conditions to promote lignin valorization. Furthermore, it provides potentially general strategies for enabling visible light-driven LMCT-mediated photocatalysis in related organic transformations.

## Abstract 2

LMCT-mediated selective cleavage of β-O-4 lignin model compounds under visible light.

## Graphical abstract

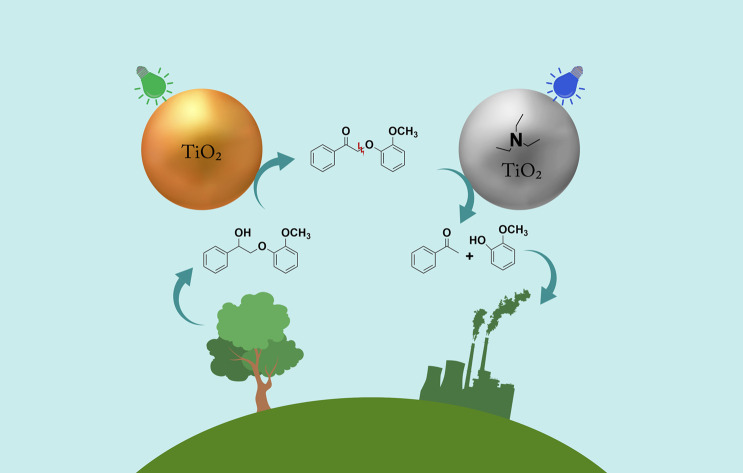

**Green foundation**

1. Our work advances the field of green chemistry through the development of a new LMCT-mediated catalytic method for the visible light valorization of lignin model compounds. Our approach addresses challenges in sustainable catalysis and mild reaction conditions in lignin valorization by avoiding toxic solvents, costly photocatalysts and UV light.

2. This achievement is highlighted through the high selectivity (up to 100%) to the target fragmentation products and high conversion of chosen model substrates and is further accentuated through acceptable model substrate conversion in one pot with a dual wavelength switching strategy by utilizing a broader range of visible light.

3. The photocatalytic reductive cleavage of naturally derived lignin molecules remains a significant challenge due to its poor solubility in various solvents, dark color, complex structure and diverse functional groups, which, if mitigated, could elevate the reaction's sustainability and applicability.

## Introduction

**Fig. 1:**
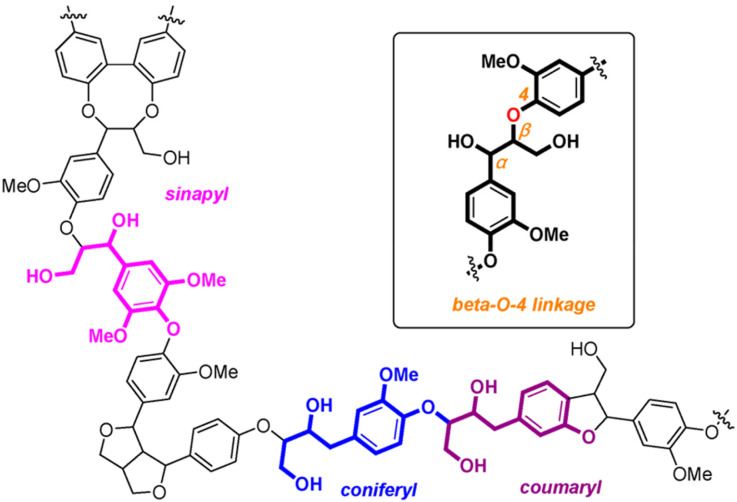
Representative structure of lignin and the β-O-4 linkage in lignin (reprinted an open access article figure^6^).

The depletion of fossil fuel reserves and the environmental problems associated with the massive consumption of fossil fuel resources have become a driving force to explore an alternative, sustainable source of energy and chemical feedstocks.^1^ Biomass is a highly abundant and promising renewable resource to produce fuels, chemicals, and functional materials. In the past decade, biomass valorization has gained significant attention for producing industrially relevant value-added chemicals.^2^ Lignin, one of the major fractions (15–35 wt%) of plant biomass, is the most abundant renewable source of aromatics in nature. It is a highly functionalized polymer (Fig. 1) made up of repeating monolignol subunits (coniferyl alcohol, sinapyl alcohol and *p*-coumaryl alcohol), connected by C–O (β-O-4, α-O-4, and 4-O-5) and C–C (β-1, β–β, β-5, and 5–5′) linkages.^3–6^

Approximately 100 million tons of lignin are produced annually as a byproduct of the pulp and paper industry and bioethanol industry worldwide.^7,8^ It is estimated that the annual lignin production will increase to 225 million tons by 2030. However, 98% of the lignin waste produced is simply burned as low-quality fuel to generate heat.^8^ Presently, only 2% of the lignin is used to produce functional materials and chemicals (like surfactants, adhesives, and dispersants),^9^ owing to its inherent heterogeneity and challenging depolymerization.^7^ The valorization of lignin offers a green and sustainable way to produce high-value, low molecular weight aromatic compounds. The key challenge in lignin valorization is the selective cleavage of the C–C and C–O bonds connecting the monolignol units in the lignin structure without inducing additional crosslinks or degradation. The β-O-4 bond is the most predominant linkage in lignin, accounting for 43–65% of all linkages.^10–13^ Therefore, the selective scission of the β-O-4 linkage is crucial for the efficient transformation of lignin into value-added chemicals.

**Scheme 1:**
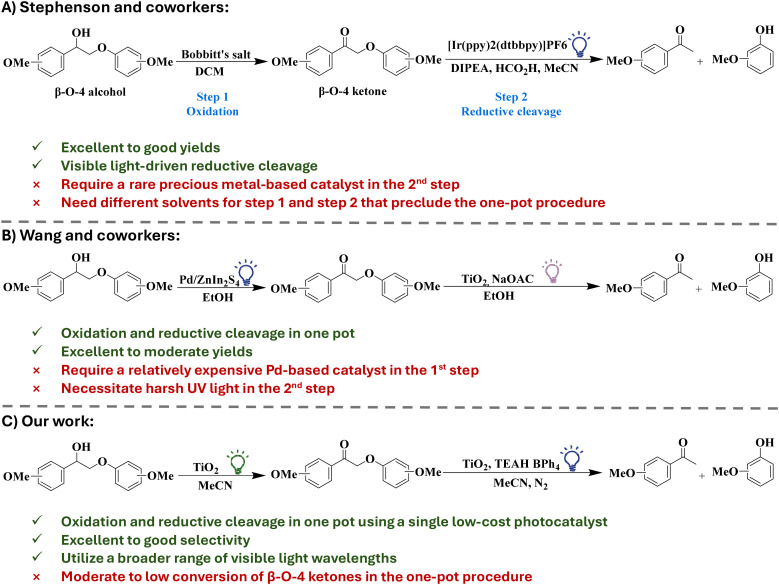
Strategies for the photocatalytic selective cleavage of β-O-4 lignin model compounds to value-added aromatic compounds.

Numerous catalytic methods, including acid/base-catalysis,^14,15^ oxidative catalysis,^16^ and reductive or redox neutral approaches,^17,18^ have been explored for the conversion of lignin model compounds to value-added aromatic compounds. In view of the complex structure of lignin, most of the studies have used representative β-O-4 lignin model compounds (specifically dilignols) instead of native lignin to explore the potential transformation of lignin into value-added products.^5,19–22^ Computational studies have shown that the oxidation of benzylic C_α_–OH bonds in lignin model compounds to the corresponding ketones reduces the bond dissociation energy of the nearby C_β_–O bond from 69 to 56 kcal mol^−1^.^23^ Based on the findings of the theoretical studies, a two-step approach has been widely adopted for β-O-4 cleavage in lignin transformation, where the oxidation of benzylic C_α_–OH to C_α_=O (respective ketone) is followed by the scission of the C_β_–O linkage (Scheme 1).^12,24,25^

Stephenson and coworkers (Scheme 1A) reported Bobbitt's salt-mediated benzylic oxidation of C_α_–OH to C_α_=O, followed by reductive cleavage of the C_β_–O bond using an iridium-based photoredox catalyst [4,4′-bis(1,1-dimethylethyl)-2,2′-bipyridine-*N*1,*N*1′]bis[2-(2-pyridinyl-*N*)phenyl-C]iridium(iii) hexafluorophosphate under visible light.^24^ This catalytic system is selective in the cleavage of β-O-4 bonds in several lignin model substrates and shows good yields (>80%) of target β-O-4 fragmentation products.^24^ However, the high cost of the iridium-based photocatalyst limits its suitability on an industrial scale. In addition, the recovery and recyclability of the catalyst and the product separation and purification are quite challenging due to the homogeneous nature of this catalytic system.

Heterogeneous catalysis can address the challenges of catalyst recovery and product separation in lignin transformation. Specifically, heterogenous photocatalysis has emerged as an efficient approach for lignin transformation as it often requires mild reaction conditions.^26–29^ Wang and coworkers (Scheme 1B) demonstrated a heterogeneous photocatalytic oxidation–reduction strategy for the cleavage of the C–O bond in β-O-4 model compounds with two distinct catalysts. The oxidation of β-O-4 alcohol to β-O-4 ketone is accomplished using Pd/ZnIn_2_S_4_ under visible light (455 nm), while titania catalyzes the reductive cleavage of the β-O-4 ketone under UV light (365 nm). The Pd/ZnIn_2_S_4_ and titania catalysts showed high selectivity (up to 90%) for ketones and target monoaromatic products, respectively.^12^ Despite that, the use of a costly, noble metal containing catalyst in the first step reduces the applicability of the method on a large scale. Moreover, the necessity of using high energy UV light in the second step introduces practical and safety challenges, which should be avoided to better align with green chemistry principles.^30,31^

The development of a visible light active and inexpensive photocatalyst for the selective cleavage of C–O bonds in lignin is a promising solution. Sun and coworkers synthesized a Ni containing CdS nanophotocatalyst for the one pot photocatalytic cleavage of a β-O-4 model compound. The prepared Ni/CdS catalyst showed excellent selectivity (>99%) for acetophenone and phenol under visible light (440–460 nm).^32^ Cossairt and coworkers were able to successfully implement Stephenson's oxidation in sequence with the photocatalytic reductive cleavage of β-O-4 ketones using CdSe quantum dots and triethylammonium salts in DCM under white light, realizing a one-pot procedure.^21^ However, the toxicity concerns of Cd-based catalysts and DCM limit the large-scale application of this approach.

In view of these facts, the development of non-toxic and inexpensive visible light active catalysts is highly desirable. Titania exhibits excellent properties suitable for large scale applications in photocatalytic lignin transformation, such as low cost, non-toxicity, chemical stability, and abundant availability. However, the major drawback of titania is its wide bandgap (∼3.2 eV), which allows it to absorb only UV light,^33^ which has a negative impact on the desired product selectivity, functional group tolerance and overall applicability of the reaction. Therefore, modifying the photo-response of titania to the visible spectrum is crucial to carry out the lignin transformation under practical, energy efficient and milder conditions.

Ligand-to-metal charge transfer (LMCT)-sensitization is one of the ways to achieve visible light activation of titania. The LMCT-sensitization involves the formation of a surface complex by the adsorbate or substrate that introduces an absorption band in the visible region.^33^ Herein, we demonstrate the one-pot, two-step, selective cleavage of the C_β_–O bond in lignin model compounds enabled by visible light active LMCT-sensitized titania for both steps. The first step involves the LMCT-mediated selective aerobic oxidation of benzylic C_α_-OH bonds in lignin model alcohols to the corresponding ketones under green light (525 nm). We hypothesized that the adsorption of β-O-4 alcohols on the surface of titania could result in the formation of a visible light-absorbing surface complex, which would enable the oxidation of benzylic alcohols to ketones under visible light. Similarly, we hypothesized that we could apply the LMCT-sensitization strategy in the second step for the reductive cleavage of the C_β_–O bond under visible light by employing a sensitizer and proton donor such as a triethylammonium salt. Our work demonstrates a promising potential of β-O-4 lignin model compound conversion to value-added aromatic compounds under mild conditions, which is a key to the advancement and economic viability of integrated biorefineries.

## Experimental

### Chemicals

Titanium(iv) isopropoxide (97+%, Sigma Aldrich), 2-propanol (99.9%, HPLC grade, Sigma Aldrich), commercial titania, P25 (99.5 %, Evonik), acetonitrile (99.9 %, HPLC grade, Fisher Chemical), methanol (99.9 %, HPLC grade, Fisher Chemical), 1,4-benzoquinone (98 %, Sigma Aldrich), silver nitrate (99.8 %, Sigma Aldrich), 2-phenoxy-1-phenylethanol (AmBeed) sodium fluoride (99 %, Acros Organics), water (HPLC grade, Fisher Chemical), ethanol (>99.5%, HPLC grade, Sigma Aldrich), triethylammonium chloride (>99%, Sigma Aldrich), sodium tetraphenylborate (>99.5%, Thermo Scientific Chemicals), sodium hexafluorophosphate (98%, Thermo Scientific Chemicals), potassium trioxalatoferrate(iii) trihydrate (Thermo Scientific Chemicals), 1,10-phenanthroline (99%, Alfa Aesar), anatase (99.7%, Thermo Scientific Chemicals) and brookite (99.9%, Sigma Aldrich) were used.

### Preparation of titania nanoparticles

Titania nanoparticles were synthesized *via* a sol–gel technique followed by a hydrothermal method adapted from a previous study.^34^ Typically, 0.129 moles of titanium(iv) isopropoxide (TTIP) were dissolved in 25 ml of 2-propanol and stirred for 2 hours at ambient temperature. Then, 1 mL of 1 M nitric acid was added to the solution under continuous stirring (400 rpm) for 5 minutes, until a gel was formed. Subsequently, 25 ml of deionized water was added to the gel and stirred for an additional 3 hours for aging. Then, the resulting precipitates were filtered, washed with deionized water twice, and dried at 110 °C in a hot air oven for 24 hours. The dried nanoparticles were transferred to a Teflon-lined stainless-steel autoclave filled with 70 mL of deionized water for hydrothermal treatment at 200 °C for 24 hours in a hot air oven. The as-prepared sample was named SGHT-200.

### Photocatalytic activity test

#### Oxidation of β-O-4 lignin model alcohol

Photocatalytic oxidation of lignin model compounds was carried out in a glass scintillation vial. Typically, 15 mL of 0.5 mM β-O-4 alcohol (0.0075 mmol) solution in acetonitrile and 45 mg of SGHT-200 were charged into the photoreactor. The photoreactor was covered with a sleeve stopper (14 × 20, VWR), sealed with the parafilm and placed in an EvoluChem PhotoRedOx Box™ (Hepato Chem). The suspension was stirred in the dark for 1 hour to attain equilibrium. Then the photoreactor was irradiated using a Kessil PR-160L 525 nm lamp for 6 hours under continuous stirring. The aliquots were collected and filtered using nylon syringe filters (0.22 μm) to remove the catalyst. After each reaction cycle, the used catalyst was collected, washed twice with acetonitrile, dried at 110 °C and then used in the next run using a fresh substrate.

#### Reductive cleavage of a β-O-4 lignin model ketone

In a typical run, a glass scintillation vial was loaded with 15 mL of 0.5 mM solution of β-O-4 ketone (0.0075 mmol) in acetonitrile, 0.075 mmol of triethylammonium tetraphenylborate, [TEAH BPh_4_] and SGHT-200 (45 mg, 3 g L^−1^). The vial was covered with a sleeve stopper, sealed with parafilm and placed in an EvoluChem PhotoRedOx Box™ (Hepato Chem). The suspension was stirred in the dark for 1 hour to establish equilibrium. The reaction mixture was then continuously bubbled with nitrogen under blue light (Kessil PR-160L 440 nm) irradiation for 6 hours. After the reaction, the used catalyst was collected, washed twice with acetonitrile, dried at 110 °C and then used in the next run using a fresh substrate.

The reactants and products were analyzed using a Shimadzu HPLC LC2040C equipped with a Thermo Scientific C18 Column (100 mm in length, 3 mm in diameter). The oven temperature was kept at 40 °C. The flow rate was set at 0.4 mL min^−1^. The mobile phase used for the quantitative analysis of β-O-4 lignin alcohol and β-O-4 lignin ketone was acetonitrile and water with a volume ratio of 55 : 45. While the quantitative analysis of guaiacol and acetophenone was carried out using a volume ratio of acetonitrile and water of 40 : 60. The conversion and selectivity were calculated in the liquid phase according to the following formulae.
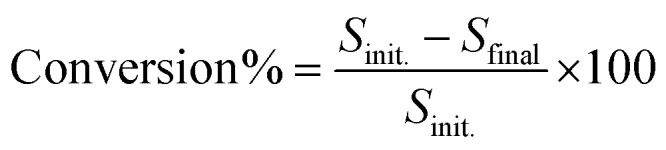

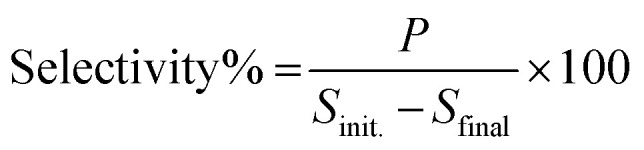
where *S*_init_ corresponds to the initial amount of substrate (mmol) and *S*_final_ refers to the final amount of substrate (mmol) after the photocatalytic reaction. *P* is the mmol of product formed after photocatalytic reaction.

### Characterization of titania nanoparticles

X-ray diffraction analysis of pristine titania and nitrogen-doped titania nanoparticles was carried out using a Bruker D8 Advance powder diffractometer with an LYNXEYE detector. Nickel filtered Cu K-alpha radiation was used with a tube voltage of 40 kV and an amperage of 40 mA. Data collection was over a 2-theta range of 5–90 degrees, with a step size of 0.02 degrees, and a collection time of 0.4 s per step. UV/visible diffuse reflectance spectroscopy (DRS) characterization of the photocatalysts was performed using an Agilent Cary 5000 UV/vis/NIR spectrometer. A PTFE reference disk was used as a standard. The IR spectrum of the photocatalysts was recorded using a Thermo Scientific spectrometer in the range of 4000–600 cm^−1^ in the transmittance mode with 32 scans and a resolution of 16 cm^−1^. X-ray photoelectron spectroscopy (XPS) measurements were performed using a Kratos Axis UltraDLD analysis system with monochromated Al Kα (*hν* = 1487 eV) X-ray source (15 kV, 10 mA). The survey scans were acquired with a pass energy of 160 eV and a step size of 1 eV. The high-resolution core level scans were acquired with a pass energy of 20 eV and a step size of 0.1 eV. Detailed information on the synthesis of substrates, sample preparation for photocatalysts characterization and control experiments is provided in the ESI.†

## Results and discussion

### Characterization of titania nanoparticles

The crystallographic features of lab-synthesized titania (SGHT-200) and commercial titania (P25) have been studied using XRD analysis (Fig. S1†). The XRD analysis of SGHT-200 shows that it consists of anatase and brookite phases at a ratio of 79 : 21 with crystal sizes of 11 and 8 nm, respectively (entry 1, Table S1†). P25 is composed of anatase and rutile phases at a ratio of 87 : 13 with crystal sizes of 25 and 42 nm, respectively (entry 2, Table S1†). The specific surface area of SGHT-200 and P25 samples was determined by the Brunauer–Emmett–Teller (BET) method. SGHT-200 exhibits a type IV isotherm with an H2 hysteresis loop (Fig. S2†), which is characteristic of a mesoporous material with ink-bottle shaped pores.^35^ In contrast, P25 shows a type II isotherm with an H3 hysteresis (Fig. S2†) that indicates slit-shaped pores. We found that SGHT-200 exhibits a higher specific surface area (109 m^2^ g^−1^) compared to P25 (46 m^2^ g^−1^).

**Fig. 2:**
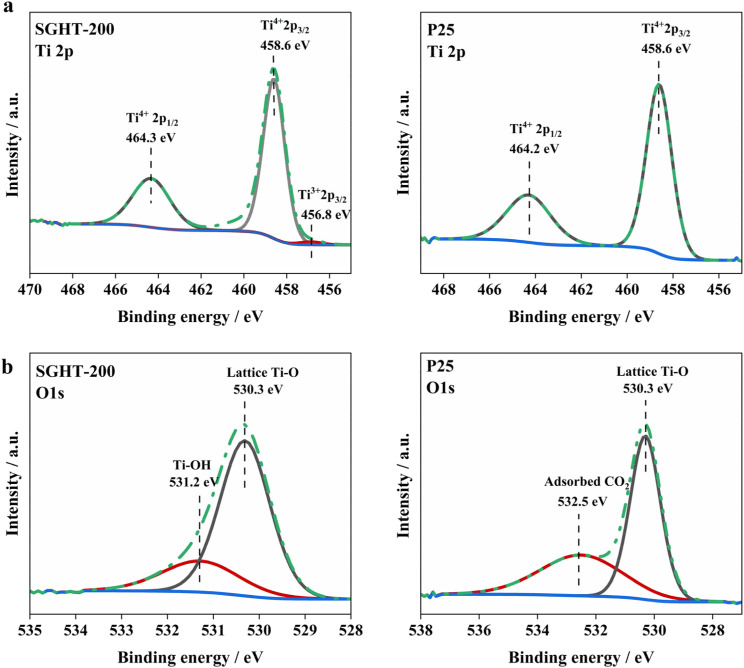
XPS (a) Ti 2p and (b) O 1s spectra of lab-synthesized titania (SGHT-200) and commercial titania (P25).

XPS analysis has been performed to investigate the surface chemistry of SGHT-200 and P25. The Ti 2p core level spectrum of SGHT-200 deconvolutes into three peaks (Fig. 2a); the peaks centered at binding energies of 458.6 and 464.3 eV correspond to Ti^4+^ 2p_3/2_ and Ti^4+^ 2p_1/2_, respectively, while the peak appearing at 456.8 eV relates to Ti^3+^ 2p_3/2_. P25 exhibits similar binding energies for Ti^4+^; however, no Ti^3+^ signal was detected in P25 (Fig. 2a). The O 1s spectrum of SGHT-200 shows two main peaks, the peak appearing at 530.3 eV (Fig. 2b), assigned to the Ti–O bond in the titania lattice,^36,37^ and the signal observed at 531.2 eV (Fig. 2b), related to the Ti–OH bonds in titania.^37^ The O 1s spectrum of P25 (Fig. 2b) exhibits a peak at 532.5 eV for the C–O bond due to adsorbed CO_2_,^38^ in addition to a lattice oxygen peak (530.3 eV).

Next, we measured the DRS UV-Visible absorption spectra of SGHT-200 and P25. SGHT-200 showed absorption in the UV region (Fig. S3†), which is typical of pristine titania. The band gap of SGHT-200 and P25 estimated through the Kubelka–Munk function was ∼3.2 eV (Fig. S4†). The transmission electron microscopy (TEM) images (Fig. S5a and b†) showed that SGHT-200 and P25 nanoparticles were highly aggregated and exhibited variable sizes and shapes. SGHT-200 showed an average particle size of 11 nm (Fig. S5c†). However, P25 exhibited a greater average particle size of 23 nm (Fig. S5d†). The TEM results were in accordance with the crystal sizes calculated from the XRD patterns.

### Selective oxidation of β-O-4 alcohols

We hypothesized that the adsorption of β-O-4 alcohols on the surface of titania (SGHT-200) should form a visible light-absorbing LMCT-complex, which can enable the aerobic oxidation of benzylic alcohols (C_α_–OH) to ketones (C_α_=O) under visible light irradiation. To probe the activity of LMCT-sensitized SGHT-200 for the selective oxidation of benzylic alcohols to ketones, a photocatalytic experiment was carried out for the selective oxidation of our model β-O-4 alcohol, 2-(2-methoxyphenoxy)-1-phenylethanol (PPE), to 2-(2-methoxyphenoxy)-1-phenylethanone (PPEn) under green light. We found that LMCT-sensitization of SGHT-200 by PPE exhibited high activity for the oxidation of PPE (entry 3, Table 1). After 6 hours of irradiation under green light, 95% PPE conversion was observed with excellent selectivity (99%) for PPEn with an optimized catalyst loading (3 g L^−1^, Fig. S6†). Commercial titania (P25), which is considered as a benchmark in heterogeneous photocatalysis, was also tested for the selective oxidation of PPE to PPEn under green light. Commercial titania (P25) also showed high selectivity (93%) for PPEn (entry 4, Table 1); however, the activity of P25 was relatively low (38% PPE conversion) for this oxidation reaction. The lower activity of P25 may arise from the lower specific surface area of P25 (46 m^2^ g^−1^) compared to SGHT-200 (109 m^2^ g^−1^). Alternatively, SGHT-200 also showed a different phase composition compared to P25, which warranted further study. To investigate the effect of phase composition, the partial oxidation of PPE has been carried out using commercial anatase, commercial brookite, and a physical mixture of brookite and anatase. All three of these catalysts showed very low activity (entries 5–7, Table 1) which is attributed to the low specific surface area of anatase (34 m^2^ g^−1^) and brookite (28 m^2^ g^−1^). These results suggest that the specific surface area of titania photocatalysts plays a larger role than phase composition in achieving high activity for β-O-4 alcohol oxidation.

**Table 1:** Photocatalytic selective oxidation of 2-(2-methoxyphenoxy)-1-phenylethanol (PPE) to 2-(2-methoxyphenoxy)-1-phenylethanone (PPEn) under green light (525 nm)

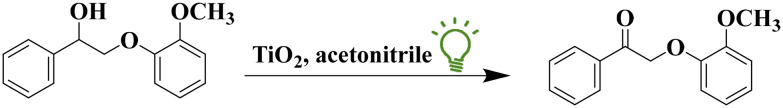					
Entry	Photocatalyst	*hν* (nm)	Additive	PPE conversion (%)	PPEn selectivity (%)
1	None	525	None	1	0
2	SGHT-200	Dark	None	0	0
3	SGHT-200	525	None	95	99
4	Commercial titania (P25)	525	None	38	93
5	Anatase	525	None	2	0
6	Brookite	525	None	9	49
7	Anatase : Brookite	525	None	6	48
8	SGHT-200-C-600	525	None	10	98
9	F-SGHT-200	525	None	2	0
10	SGHT-200	525	N_2_	0	0
11	SGHT-200	525	1,4-Benzoquinone	32	19
12	SGHT-200	525	AgNO_3_	65	81
13^a^	SGHT-200	525	None	5	0

a Solvent (ethanol), light source: Kessil PR160 L LED, 525 nm (max 44 W) and average intensity: 399 mW cm^−2^ (measured from 1 cm distance).

Next, we studied the effect of irradiation wavelength on the photocatalytic performance of SGHT-200 in the selective oxidation of PPE to PPEn (Table 2). We observed 98% PPE conversion after 4 hours of irradiation under blue light (440 nm). However, the selectivity of PPEn was slightly reduced (entry 2, Table 2) at a wavelength of 440 nm, which is related to the formation of benzaldehyde in addition to PPEn due to C–C bond cleavage under relatively high energy blue light irradiation. The slightly higher wavelength blue light source (456 nm) showed similar selectivity for PPEn (entry 3, Table 2).

**Table 2:** Effect of the irradiation wavelength on the photocatalytic performance of SGHT-200 in the selective oxidation of 2-(2-methoxyphenoxy)-1-phenylethanol (PPE) to 2-(2-methoxyphenoxy)-1-phenylethanone (PPEn)

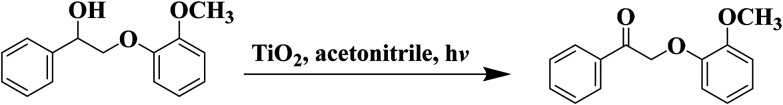					
Entry	Photocatalyst	*hν* (nm)	Additive	PPE conversion (%)	PPEn selectivity (%)
1	SGHT-200	525	None	95	99
2^a^	SGHT-200	440	None	98	81
3	SGHT-200	456	None	96	80

a Irradiation time (4 hours) Light source: Kessil PR160 L LED, 440 nm (max 44 W) and 456 nm (max 50 W), and average intensity: 399 mW cm^−2^ (measured from a distance of 1 cm).

The scope of the oxidation of β-O-4 alcohols to their corresponding ketones was studied under optimal conditions using SGHT-200. The β-O-4 alcohols with a methoxy group (–OCH_3_) on one or both benzene rings (entries 2 and 3, Table 3) showed higher conversion compared to the β-O-4 alcohols lacking methoxy groups (entry 1, Table 3). The presence of methoxy groups on the benzene ring gives the system more electrons which further enhances the activity of the LMCT-complex. However, the selectivity for respective β-O-4 ketones remains high (>95%) for all the three substrates (entries 1–3, Table 3). Additionally, we performed time-course experiments to investigate the selectivity of β-O-4 ketones over the course of 6 hours of irradiation. For all three substrates, the selectivity for β-O-4 ketones remains high (>95%) over a series of time points (Fig. S7–S9†). This indicates that relatively mild green light prevents the degradation of the substrates and targets β-O-4 ketones, ensuring high selectivity.

**Table 3:** Photocatalytic selective oxidation of β-O-4 alcohols to β-O-4 ketones under green light (525 nm)

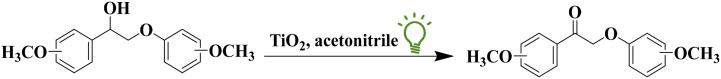			
Entry	Photocatalyst	Substrate conversion (%)	Product selectivity (%)
1	SGHT-200	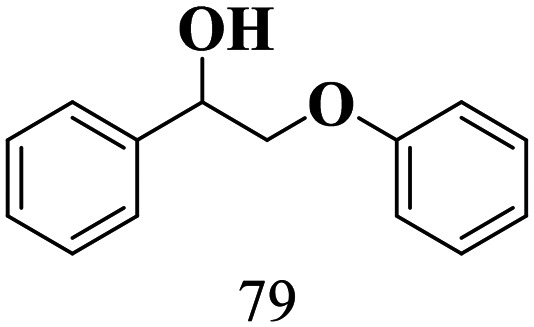	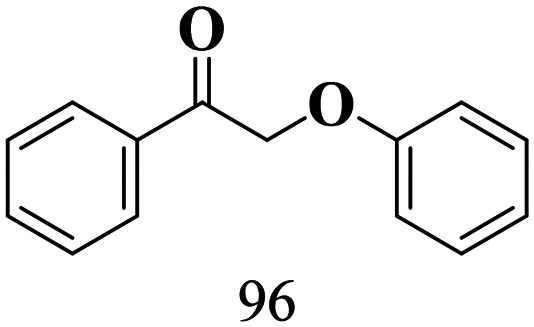
2	SGHT-200	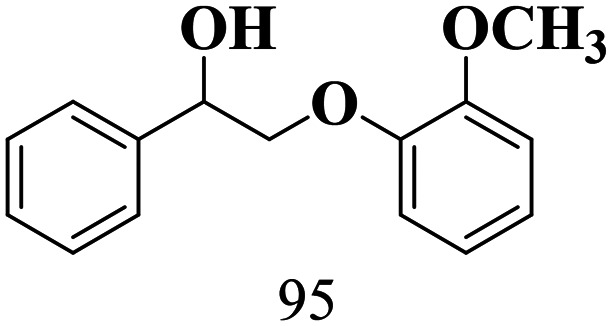	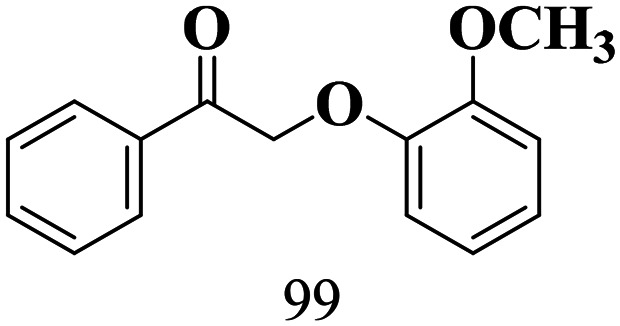
3	SGHT-200	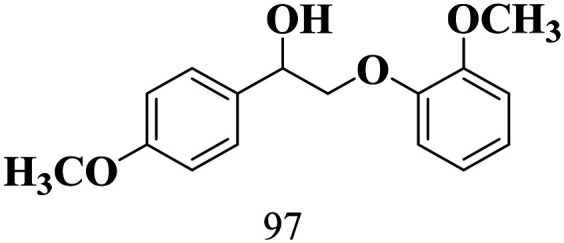	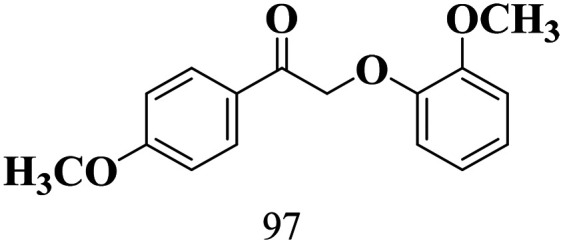

**Fig. 3:**
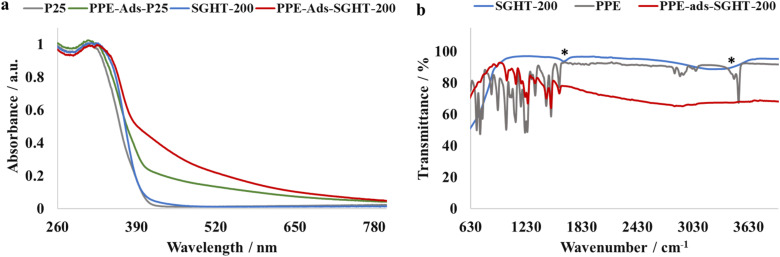
(a) DRS-UV-Visible absorption spectra of titania and PPE adsorbed titania samples. (b) IR spectra of titania and PPE adsorbed titania samples. Note: *O–H stretching and bending vibrations.

The recyclability of SGHT-200 was assessed in the selective oxidation of PPE to PPEn under green light. As shown in Fig. S10†, SGHT-200 significantly lost its activity in the third cycle. We carried out detailed characterization of SGHT-200 after each cycle to investigate the reduced activity of the catalyst. The XRD analysis (Fig. S11 and Table S2†) shows that SGHT-200 exhibits similar crystal size and phase composition (entries 1–6, Table S2†) after multiple runs, which indicates that SGHT-200 retained its crystallographic features after reuse. However, we observed a slight reduction in the specific surface area of SGHT-200 after reuse (entries 1–6, Table S2†). This may be associated with the initially adsorbed PPE on the surface, which occupies the surface sites and reduces the available surface area. Sun and coworkers also observed a slight reduction in the surface area of TiO_2_ used in organic wastewater treatment due to carbon accumulation on the surface.^39^ We observed that the specific surface area, which correlates with the number of adsorption sites of the photocatalyst, plays a crucial role in the activity of SGHT-200 for the selective oxidation of PPE. It is presumed that the attenuation of activity for SGHT-200 in subsequent cycles may arise from a reduction in the number of adsorption sites (OH groups) for PPE. The PPE initially bound to SGHT-200 may form a stable surface complex, which is resistant to desorption while washing the catalyst. This may reduce the number of adsorption sites for the incoming PPE molecules. To test this hypothesis, we performed an IR analysis of SGHT-200 washed with acetonitrile and water after each cycle (Fig. S12 and S13†). SGHT-200 used in multiple runs showed weak bands in 1209–1240 cm^−1^ and 1440 cm^−1^ regions (Fig. S12 and S13†) that are characteristic of PPE (Fig. 3b). This suggests that PPE forms a stable complex on the surface of titania, which is not easily disrupted by washing with solvents. This is also indicated by the color of SGHT-200 which remains yellow even after washing (Fig. S14†). Additionally, the surface OH groups of titania that are considered crucial for the adsorption of PPE and LMCT-mediated oxidation of PPE are not regenerated even after washing with water (Fig. S13†). This makes the reuse of SGHT-200 for multiple cycles challenging.

The quantum yield of the β-O-4 ketone (PPEn) has been measured by standard potassium ferrioxalate actinometry (Fig. S15†) to study the efficiency of photocatalytic oxidation of a β-O-4 alcohol (PPE) to a β-O-4 ketone (PPEn) under green light. The quantum yield (*Φ*) achieved for the β-O-4 ketone (PPEn) after 6 hours of irradiation with SGHT-200 was 2%. The commercial titania (P25) showed 1% quantum yield for PPEn production after 6 hours of irradiation. The low quantum yields in heterogenous photocatalytic reactions are mainly due to the scattering and reflection of irradiation with a solid suspended photocatalyst.

#### Ligand-to-metal charge transfer complex formation

We postulated that β-O-4 alcohols adsorb on the surface of SGHT-200 and form a LMCT-complex. The resulting complex can improve the visible light absorption of SGHT-200 by introducing a new absorption band in the visible light region. To confirm that the activity of SGHT-200 under visible light is related to the formation of a visible light absorbing LMCT-complex, the effect of PPE adsorption on the absorption spectrum of SGHT-200 has been investigated. Titania typically shows absorption only in the UV region. The DRS-UV-Visible absorption spectrum of β-O-4 alcohol (PPE) adsorbed SGHT-200 (Fig. 3a) shows that the adsorption of PPE on SGHT-200 results in a substantial increase in the absorption of visible light (up to 700 nm). The formation of a surface complex is also indicated by a change in the color of SGHT-200 from white to yellow (Fig. S16†). Commercial titania (P25) also showed improvement in the absorption of visible light upon PPE adsorption (Fig. 3a). However, SGHT-200 displayed relatively pronounced absorption in the visible region of the spectrum, which is crucial in promoting the photocatalytic oxidation of a β-O-4 alcohol to a β-O-4 ketone under green light.

To gain further insight into surface complex formation, PPE, SGHT-200 and PPE adsorbed SGHT-200 (PPE-ads-SGHT-200) were analyzed by IR spectroscopy. As seen in Fig. 3b, the PPE adsorbed titania sample (PPE-ads-SGHT-200) exhibits bands characteristic of PPE in the 1000–1600 cm^−1^ region, while SGHT-200 lacks bands in that specific region. Interestingly, the band corresponding to the O–H stretching vibration in PPE and SGHT-200 (surface hydroxyl groups) was not observed in the PPE adsorbed titania sample (PPE-ads-SGHT-200), which we hypothesize correlates with the coordination of oxygen (alkoxide group) to the Ti site, accompanied by the loss of a proton. These results are consistent with the previous studies carried out on LMCT-sensitization of titania by 5-hydroxymethylfurfural;^34^ similar IR spectral features have been observed when 5-hydroxymethylfurfural was adsorbed on the surface of titania. It was reported that the adsorption of 5-hydroxymethylfurfural on titania is dissociative in nature due to the lack of an observable O–H stretching vibration, we propose a similar interaction between PPE and SGHT-200.

To explore the role of surface hydroxyl groups in visible light absorbing LMCT-complex formation, SGHT-200 was calcined under static air at a high temperature (600 °C) to remove surface hydroxyl groups. The IR spectra (Fig. S17†) of the calcined titania sample (SGHT-200-Cal-600) showed that the band for the O–H stretching vibration at 3200–3400 cm^−1^ and bending vibrations at 1641 cm^−1^ disappeared after calcination. As anticipated, the activity of titania (SGHT-200-Cal-600) was substantially reduced after calcination for the partial oxidation of PPE to PPEn (entry 8, Table 1). After 6 hours of irradiation, 10% PPE conversion was achieved; however, the selectivity of PPEn was not affected after calcination (entry 8, Table 1). This indicates that hydroxyl groups are crucial for LMCT-complex formation and visible light activity.

To further corroborate the role of surface hydroxyl groups in LMCT-complex formation, surface fluorination of SGHT-200 was carried out to replace the surface hydroxyl groups with fluorine. XPS measurement was performed to confirm the substitution of hydroxyl groups with fluorine. The F 1s spectrum (Fig. S18†) of the fluorinated titania sample (F-SGHT-200) showed two peaks at binding energies of 681.7 and 683.7 eV. These peaks are commonly observed in fluorinated titania originating from surface-bound Ti–F groups.^40^ Moreover, the O 1s spectrum of F-SGHT-200 did not show a signal for the Ti–OH bond (Fig. S19†) which indicates the substitution of OH groups with fluorine. As hypothesized, surface-fluorination diminished the activity of titania (entry 9, Table 1) for the partial oxidation of PPE (2% conversion). This signifies that hydroxyl groups are critical for surface-complex formation and consequent visible light photocatalytic activity of titania for the selective oxidation of PPE to PPEn.

#### Mechanistic studies

To identify the key species involved in the photocatalytic oxidation of β-O-4 alcohol to β-O-4 ketone, the selective oxidation of PPE was carried out in the presence of different additives. It was observed that bubbling N_2_ in the reaction medium completely inhibits the oxidation of PPE (entry 10, Table 1). This indicates that O_2_ is critical and likely the terminal oxidant for the partial oxidation of PPE. The addition of 1,4-benzoquinone as a superoxide radical anion (O_2_˙^−^) scavenger (entry 11, Table 1) drastically reduced the activity of SGHT-200. The conversion of PPE and selectivity for PPEn dropped to 32% and 19%, respectively. This further corroborates that O_2_ is crucial for oxidation and superoxide radical anions (O_2_˙^−^) are likely involved in the partial oxidation of PPE to PPEn. Furthermore, introducing silver(i) nitrate into the reaction medium as an electron acceptor significantly reduced the conversion of PPE (65%) and the selectivity of PPEn (81%) by SGHT-200 (entry 12, Table 1). It is proposed that silver(i) ions in solution oxidize Ti(iii), preventing the formation of superoxide radical anions normally formed by the oxidation of Ti(iii) by O_2_.

**Fig. 4:**
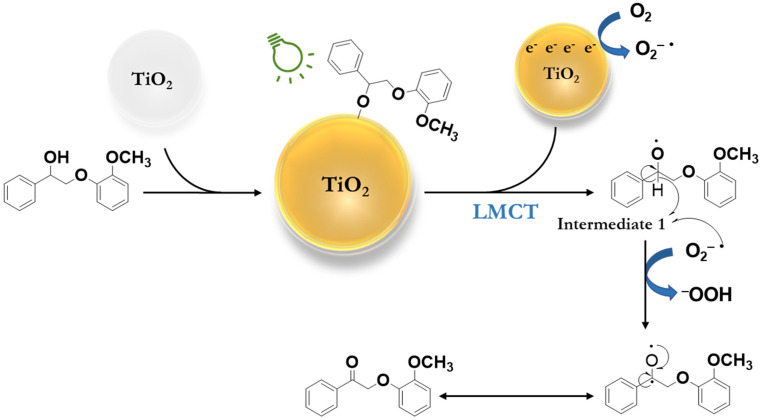
Plausible reaction mechanism of selective oxidation of a β-O-4 alcohol (PPE) to a β-O-4 ketone (PPEn) in acetonitrile under green light (525 nm).

Based on these results, a plausible reaction mechanism has been proposed for the selective oxidation of β-O-4 alcohols (Fig. 4) under green light. It begins with the adsorption of a β-O-4 alcohol (PPE) on the surface of titania, where the hydroxyl group of the β-O-4 alcohol (PPE) is deprotonated and directly bound to the Ti site, resulting in LMCT-complex formation. The green light irradiation of the LMCT-complex transports an electron into the conduction band of the titania and forms Intermediate 1. The naturally dissolved oxygen in the reaction medium is reduced to a superoxide radical anion (O_2_˙^−^) by accepting an electron from the conduction band of titania. The superoxide radical anion (O_2_˙^−^) abstracts a hydrogen atom from Intermediate 1 to form PPEn. We proposed that the hydroperoxide anion deprotonates the β-O-4 alcohol and generates hydrogen peroxide as a side product.

### Selective cleavage of a β-O-4 ketone in acetonitrile

We demonstrated that the sensitization of titania by a β-O-4 alcohol is suitable for the selective oxidation of a β-O-4 alcohol to a β-O-4 ketone. We also envisioned being able to carry out the reductive cleavage of β-O-4 ketones under comparable conditions (*e.g.* with acetonitrile as a solvent), with the goal of developing a one-pot transformation for both the oxidation and reductive cleavage steps. SGHT-200 was found to be inactive for the reductive cleavage of PPEn to guaiacol and acetophenone under blue light (440 nm) in acetonitrile (entry 1, Table 4). However, when the experiment is carried out in the presence of triethylammonium salts (triethylammonium tetraphenylborate, [TEAH BPh_4_] or triethylammonium hexafluorophosphate, [TEAH PF_6_]), which can act as proton donors and photosensitizers, SGHT-200 successfully catalyzes the reductive cleavage of PPEn in acetonitrile, with moderate to high selectivity for the target products (entries 2 and 3, Table 4). The optimized catalyst loading of 3 g L^−1^ of SGHT-200 showed full conversion for PPEn after 4 hours of irradiation (Fig. S20†). Commercial titania (P25) was also found to be active for the reductive cleavage of PPEn in the presence of TEAH BPh_4_ (entry 4, Table 4); however, it exhibited much less activity than SGHT-200. The higher activity of SGHT-200 may be related to its higher specific surface area than P25 (entries 1 and 2, Table S1†). We then studied the activity of SGHT-200 under longer wavelength blue light (456 nm and 467 nm) and green light (525 nm). SGHT-200 achieved full conversion of PPEn under 456 nm and 467 nm (entries 2 and 3, Table 5), but no PPEn conversion was observed under green light (entry 4, Table 5). The relatively lower selectivity of guaiacol might be related to the slow desorption of guaiacol from the catalyst surface. The GC MS results showed that no other side products were observed for the reductive cleavage of PPEn (Fig. S21–S25†).

**Table 4:** Photocatalytic reductive cleavage of β-O-4 ketones under blue light (440 nm) in the presence of triethylammonium tetraphenyl borate (TEAH BPh_4_)

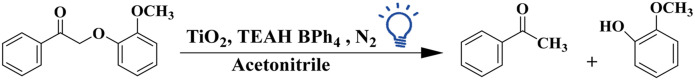						
Entry	Photocatalyst	*hν* (nm)	Additive	PPEn conversion (%)	Acetophenone selectivity (%)	Guaiacol selectivity (%)
1	SGHT-200	440	None	8	0	0
2	SGHT-200	440	TEAH PF_6_	43	92	41
3	SGHT-200	440	TEAH BPh_4_	100	88	73
4	P25	440	TEAH BPh_4_	29	100	73
5	F-SGHT-200	440	TEAH BPh_4_	5	100^b^	85
6	SGHT-200-C-600	440	TEAH BPh_4_	7	100^b^	63
7	SGHT-200	440	TEAH BPh_4_ + AgNO_3_	2	100^b^	89
8^a^	SGHT-200	440	TEAH BPh_4_	10	100^b^	82
9	SGHT-200	440	TEAH BPh_4_ + DIPEA	100	84	70

a Experiment carried out without N_2_ flow.

b The selectivity of acetophenone exceeds 100%. This inaccuracy in the selectivity value is associated with the low conversion of the substrate and may be related to solvent evaporation under nitrogen flow or an artifact. This has been verified by GC MS analysis (Fig. S21–S25†), which shows that the compounds detected other than guaiacol and acetophenone in the reaction medium are related to the degradation of TEAH BPh_4_, contamination from the septum that is used to cover the photoreactor and column bleed from the instrument.

**Table 5:** Effect of the irradiation wavelength on the photocatalytic performance of SGHT- 200 in the reductive cleavage of 2-(2-methoxyphenoxy)-1-phenylethanone (PPEn)

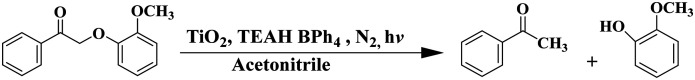						
Entry	Photocatalyst	*hν* (nm)	Additive	PPEn conversion (%)	Acetophenone selectivity (%)	Guaiacol selectivity (%)
1	SGHT-200	440	TEAH BPh_4_	100	88	73
2	SGHT-200	456	TEAH BPh_4_	100	88	68
3	SGHT-200	467	TEAH BPh_4_	100	73	54
4	SGHT-200	525	TEAH BPh_4_	0	0	0

The scope of β-O-4 ketones in reductive cleavage has been studied under the same conditions using SGHT-200 in the presence of TEAH BPh_4_. As shown in Table 6, SGHT-200 showed high conversion for the reductive cleavage of three different β-O-4 model ketones with good to excellent selectivity for the target products. The presence or absence of a methoxy substituent on the benzene ring did not drastically affect the reactivity, as illustrated by high conversion (>80%) observed for all three tested substrates. This provides valuable insight into the selective cleavage of β-O-4 model compounds under mild conditions, including the influence of methoxy substituents on the substrate. Furthermore, we noted that control reactions have shown that TEAH BPh_4_ produce phenol and other aromatic byproducts *via* C–B bond oxidative cleavage under photocatalytic conditions used for the reductive cleavage of ketones. Since the reductive cleavage of 2-phenoxy-1-phenylethanone (entry 1, Table 6) produces phenol (in addition to acetophenone), it is challenging to determine the actual selectivity for phenol in the presence of TEAH BPh_4_. However, for other substrates where this byproduct does not interfere with product quantification (*e.g.* for guaiacol, 2-methoxyphenol), good selectivity (>70%) is observed.

**Table 6:** Photocatalytic reductive cleavage of β-O-4 ketones under blue light (440 nm) in the presence of triethylammonium tetraphenyl borate (TEAH BPh_4_)

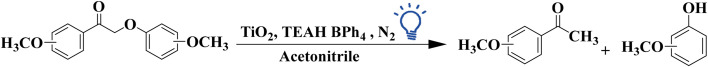				
Entry	Photocatalyst	Substrate conversion (%)	Product selectivity (%)	
1	SGHT-200	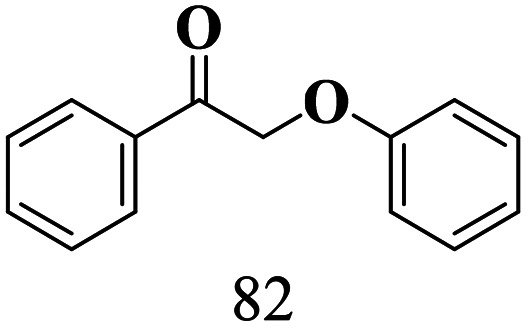	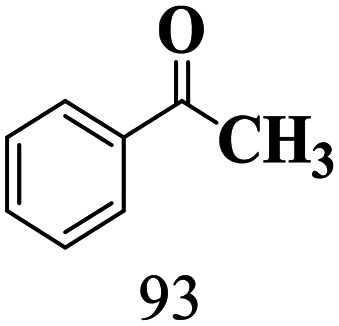	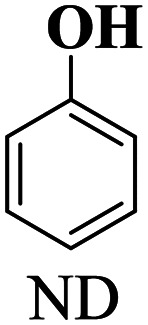
2	SGHT-200	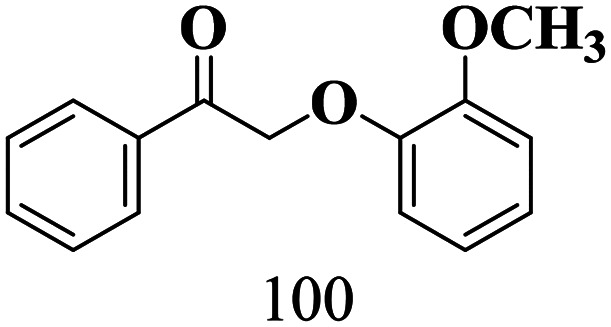	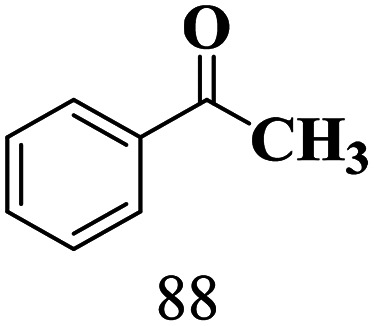	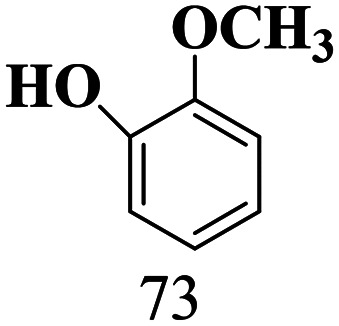
3	SGHT-200	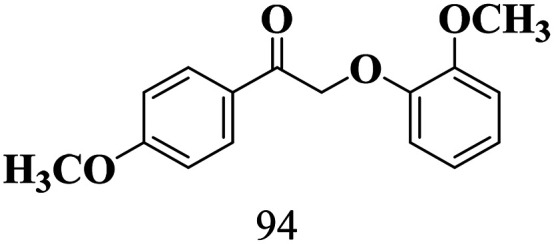	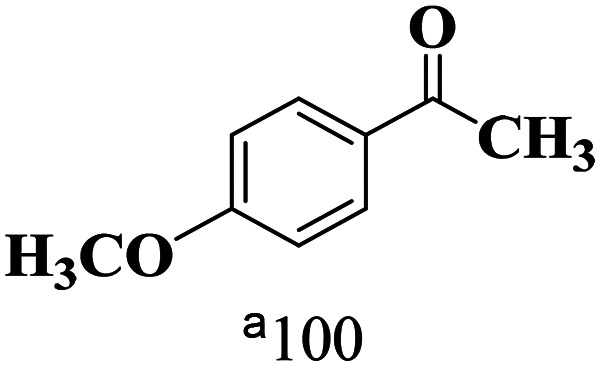	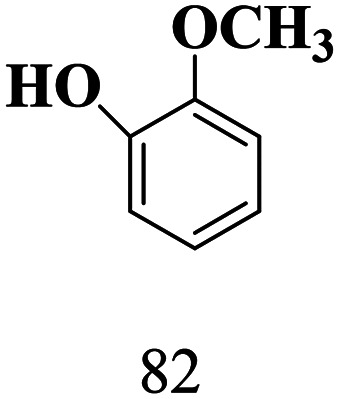

a The selectivity exceeds 100%. This inaccuracy in the selectivity value may be related to solvent evaporation or an artifact. This has been verified by GC MS analysis (Fig. S21–S25†).

The time course experiments were carried out for the reductive cleavage of three substrates to investigate the trend of selectivity for the target products over a period of time (Fig. S26–S28†). The selectivity of acetophenone remains stable (>85%) during the course of the experiment (Fig. S26–S28†), while the selectivity of guaiacol is lower in the beginning (until 2 hours) and then increases with time and remains stable for the rest of the experiment (Fig. S26–S28†). The low selectivity of guaiacol in the beginning might be related to the slow desorption of guaiacol from the titania surface. Besides that, guaiacol may readsorb over the surface of SGHT-200 through OH group interaction, which may reduce the selectivity of guaiacol.

We were not able to test the naturally derived lignin molecules in our study due to the poor solubility of low sulfonate alkali lignin and ethanosolv (type of organosolv lignin) lignin in acetonitrile. Wang and coworkers have performed cleavage of organosolv lignin in ethanol; however, the dark brown color of lignin limits the absorption of light and no products were observed after the reaction.^12^ The photocatalytic reductive cleavage of naturally derived lignin molecules remains a significant challenge due to its poor solubility in various solvents, dark color, complex structure and diverse functional groups.

The reusability of SGHT-200 was evaluated in the reductive cleavage of a β-O-4 ketone under blue light (440 nm). It was observed that SGHT-200 significantly lost its activity in the 2^nd^ cycle (Fig. S29†). Only 9% conversion has been achieved in the 2^nd^ run for the reductive cleavage of the β-O-4 ketone (PPEn); however, the selectivity of target products remains high (Fig. S29†). The loss of activity of SGHT-200 may be related to the strong adsorption of degradation products of TEAH BPh_4_ and guaiacol on the surface of titania that may block the adsorption sites (for fresh triethylamine) in the next run. To investigate the inactivity of SGHT-200, we performed the characterization of SGHT-200 after each cycle after washing with acetonitrile and water. The crystallographic features of SGHT-200 (crystal size and phase composition) did not change significantly after multiple runs (Fig. S30 and Table S3†). However, the specific surface area of the reused catalyst was slightly reduced (entries 1–4, Table S3†), this may be ascribed to the adsorption of degradation products of TEAH BPh_4_. The IR analysis (Fig. S31 and S32†) of the reused catalyst showed the presence of multiple weak bands at 1162 cm^−1^, 1245 cm^−1^ and 1438 cm^−1^ which may arise from the adsorption of guaiacol and degradation products of TEAH BPh_4_. Besides that, the O–H stretching vibration related to the surface OH groups was not observed even after washing with water (Fig. S32†). These results show that some compounds remain adsorbed over the surface of the catalyst even after washing with solvents, which is also indicated by the color of SGHT-200 which remains yellow after washing (Fig. S33†). This hinders the adsorption of triethylamine in the next run and makes the reuse of SGHT-200 in the reductive cleavage of the β-O-4 ketone (PPEn) challenging.

Additionally, we performed actinometric measurements (Fig. S34†), and the quantum yields (*Φ*) achieved for guaiacol and acetophenone in the reductive cleavage of the β-O-4 ketone by SGHT-200 were 3% and 4%, respectively under blue light (440 nm). Commercial titania (P25) showed much lower quantum yields of 1% for both guaiacol and acetophenone after 6 hours of irradiation. The low quantum yields indicate that the reaction does not proceed through a radical chain mechanism.

### Ligand-to-metal charge transfer complex formation

**Fig. 5:**
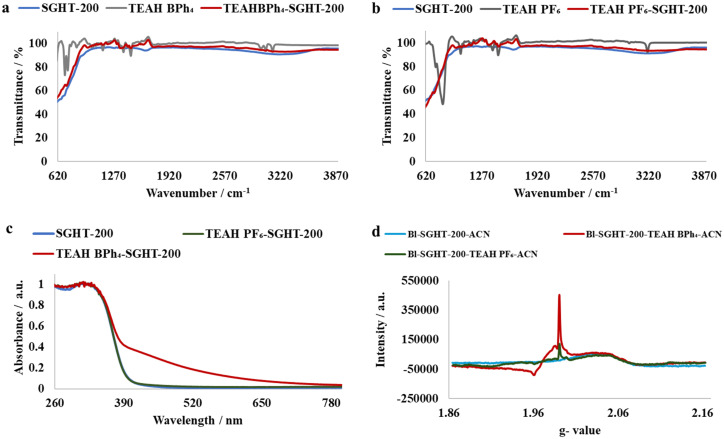
(a) IR spectra of titania, triethylammonium tetraphenylborate (TEAH BPh_4_) and titania samples exposed to TEAH BPh_4_. (b) IR spectra of titania, triethylammonium hexafluorophosphate (TEAH PF_6_) and titania samples exposed to TEAH PF_6_. (c) DRS-UV-Visible absorption spectra of pristine titania and titania samples exposed to triethylammonium salts. (d) EPR spectra of the titania suspension in triethylammonium salts in acetonitrile after blue light irradiation (440 nm).

We propose that triethylammonium salts (TEAH BPh_4_ and TEAH PF_6_) serve as visible light sensitizers for titania through the amine functional group. The triethylammonium ion can undergo deprotonation and form a complex on the surface of titania. To provide evidence for this, we performed IR analysis of SGHT-200, triethylammonium salts, and a titania sample exposed to triethylammonium salts in acetonitrile. As seen in Fig. 5a and b, the triethylamine (NEt_3_) adsorbed titania samples showed band characteristics of triethylammonium salts, while SGHT-200 lacks bands in this region. Interestingly, the band corresponding to N–H stretching vibrations was absent in the titania samples exposed to triethylammonium salts in acetonitrile, which we hypothesize correlates with the coordination of nitrogen to the Ti, accompanied by deprotonation. These results are consistent with the previous studies carried out by Scaiano and coworkers, who observed similar IR spectral features when indole (containing an N–H bond) was adsorbed over the surface of titania *via* deprotonation and formed a visible light absorbing complex.^41^

To explore further, we examined the effect of surface association of NEt_3_ on the absorption spectrum of titania. It was observed that when TEAH BPh_4_ was used as the triethylammonium source, a marked red shift occurred in the absorption spectrum of titania (Fig. 5c), while TEAH PF_6_ as the triethylammonium source did not result in any obvious change in the absorption spectrum of titania (Fig. 5c). This indicates that in addition to the amine group, the phenyl rings in BPh_4_^−^ are possibly involved in surface complex formation too, which enhances the visible light absorption. Beranek and coworkers reported that benzene can form a complex over the surface of TiO_2_ due to the interaction between π-electrons of the aromatic ring and Ti–OH and/or Ti^4+^.^42^ We propose that phenyl rings in BPh_4_^−^ interact with titania in a similar way; however, further investigation is required to test this hypothesis. The role of BPh_4_^−^ in enhancing visible light absorption of titania is also partly evident from the improved photocatalytic activity of the titania in the presence of TEAH BPh_4_ compared to TEAH PF_6_ under the same conditions (entries 2 and 3, Table 4).

To investigate the phenomenon of surface association of NEt_3_ further, and to confirm that the surface complexation of NEt_3_ results in *in situ* generation of Ti^3+^ under blue light irradiation (440 nm), EPR analysis was performed on titania samples irradiated in the presence of triethylammonium salts in acetonitrile. As seen in Fig. 5d, the titania samples irradiated in the presence of TEAH BPh_4_ and TEAH PF_6_ under inert conditions (N_2_) showed signals at *g* = 1.96 and *g* = 1.98 associated with Ti^3+^.^43,44^ While the titania samples irradiated in the absence of triethylammonium salts did not show any signal related to Ti^3+^. This suggests that the complexation of NEt_3_ is integral for Ti^3+^ generation under blue light irradiation.

We hypothesized that the surface hydroxyl groups play a critical role in the adsorption of NEt_3_ and visible light activity of titania similar to β-O-4 alcohol adsorption. The activity of surface fluorinated titania and calcined titania was assessed in β-O-4 ketone (PPEn) cleavage under blue light. Insignificant conversion has been observed for β-O-4 ketone (PPEn) cleavage by fluorinated titania (entry 5, Table 4) and calcined titania (entry 6, Table 4). This provides evidence that the surface OH groups on titania are essential for the adsorption of NEt_3_ and the formation of a visible light active surface complex for the reductive cleavage of the β-O-4 ketone (PPEn).

#### Mechanistic studies

To achieve a further understanding of the potential role of Ti^3+^ in the cleavage of β-O-4 ketone, the reductive cleavage of PPEn was performed in the presence of silver(i) salts as an electron acceptor. The addition of silver(i) cations suppressed the photocatalytic activity of SGHT-200 (entry 7, Table 4). We hypothesized that this is due to the rapid oxidation of Ti(iii) by silver(i), resulting in the substrate being unable to be reduced. Moreover, when the reaction was performed without nitrogen flow, SGHT-200 activity for PPEn conversion was drastically reduced (entry 8, Table 4). This suggests that Ti^3+^ formed is quickly oxidized to Ti^4+^ by dissolved oxygen in the reaction medium and is thus unable to participate in the reductive cleavage of PPEn. The addition of *N*,*N*-diisopropylethylamine (DIPEA) as a hole scavenger did not affect the conversion significantly (entry 9, Table 4). This indicates that holes may not be formed, as the activity of titania under blue light is ascribed to LMCT-sensitization by NEt_3_.

**Fig. 6:**
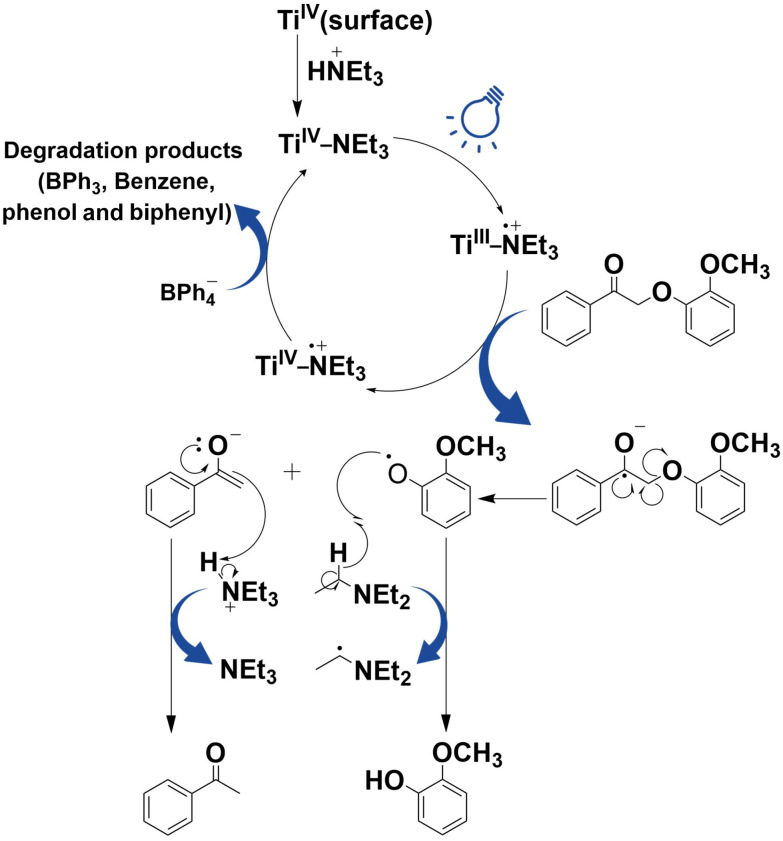
Plausible reaction mechanism of reductive cleavage of β-O-4 ketones under blue light (440 nm).

A plausible reaction mechanism for the reductive cleavage of β-O-4 ketones is proposed in Fig. 6, based on mechanistic experiments and prior studies carried out on the oxidation of amines to imine by catechol-sensitized TiO_2_.^45^ The triethylammonium ion [HNEt_3_]^+^ is deprotonated and forms a complex over the surface of titania *via* a Ti–N bond. The blue light irradiation of the surface complex transfers an electron from NEt_3_ to the conduction band of titania, while reducing Ti^4+^ to Ti^3+^. The *in situ* formed Ti^3+^ reduces the substrate to the radical anion, which is capable of undergoing beta-scission to generate a phenoxy radical and the acetophenone enolate. A hydrogen atom transfer event between triethylamine and the phenoxy radical intermediate could form guaiacol, while the protonation of the intermediate enolate by triethylammonium could furnish acetophenone. BPh_4_^−^ undergoes oxidative degradation in the process and generates numerous byproducts such as phenol, biphenyl, benzene, triphenyl borane, *etc*. based on GC-MS analysis (Fig. S24 and S25†).

We then tried to achieve the cleavage of the C_β_–O bond of β-O-4 alcohols in a one-pot, two-step process by switching the wavelength of irradiation. In the first step, β-O-4 alcohols were oxidized to β-O-4 ketones under an aerobic atmosphere and green light. Then, the reductive cleavage of β-O-4 ketones was achieved under an inert atmosphere (N_2_) and blue light in the presence of TEAH BPh_4_. As seen in Table 7, entries 1–3 show that β-O-4 alcohols were oxidized to β-O-4 ketones with high conversion in the first step; in the second step, low to moderate conversion has been observed for reductive cleavage of β-O-4 ketones with good to high selectivity for desired cleavage products. Our work demonstrates that the LMCT-sensitization of titania provides a mild two-step strategy for selective cleavage of β-O-4 model compounds in one pot without a solvent change. We expect that the development of visible light-driven LMCT-mediated strategies like the two distinct methods described here will open a new avenue in related organic transformation and biomass valorization, allowing oxidation and reduction under more selective conditions.

**Table 7:** One pot two-step photocatalytic cleavage of β-O-4 alcohols under visible light by SGHT-200

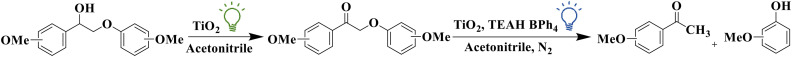					
Entry	β-O-4 alcohol conversion (%)	β-O-4 ketone selectivity (%)	β-O-4 ketone conversion (%)	Product selectivity (%)	
1	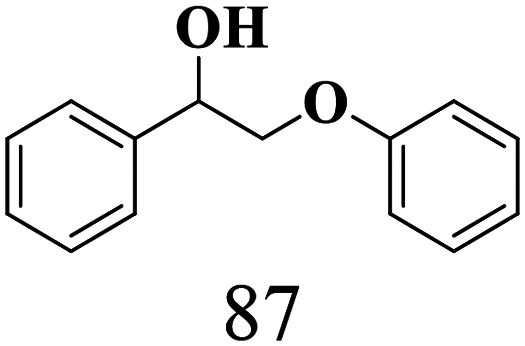	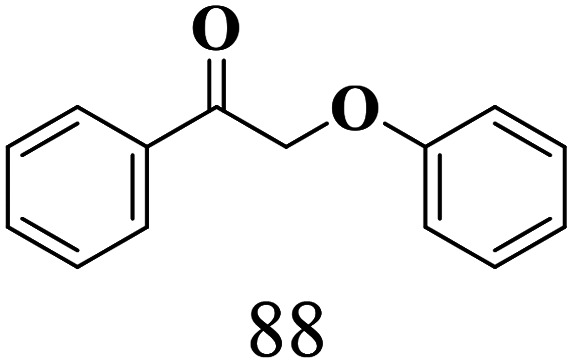	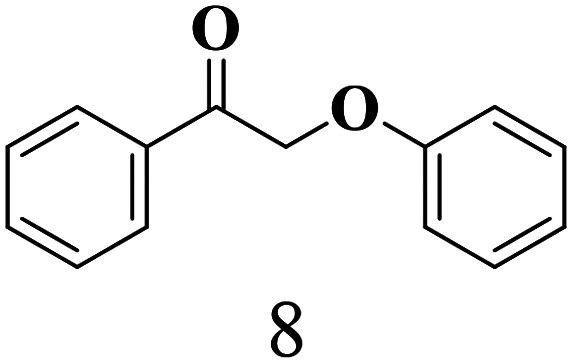	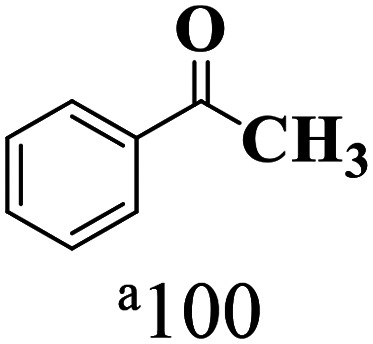	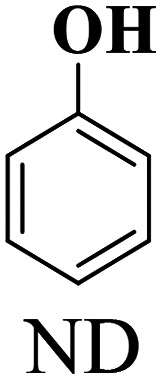
2	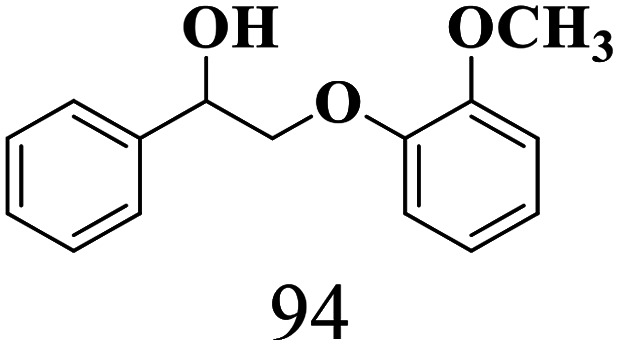	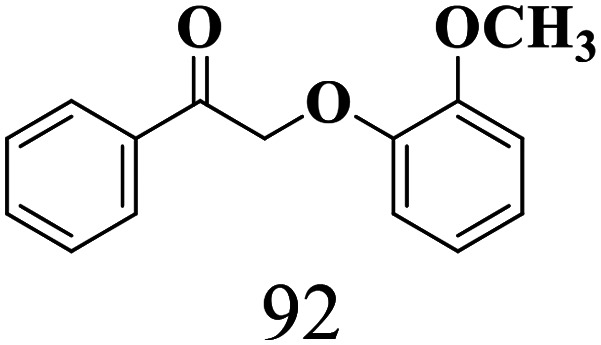	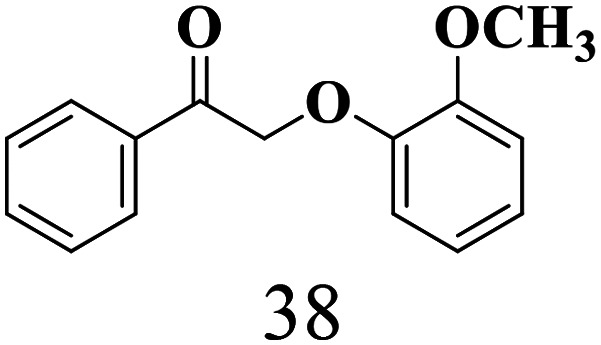	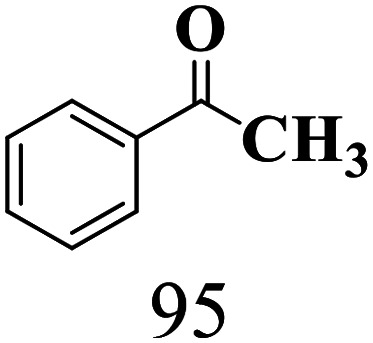	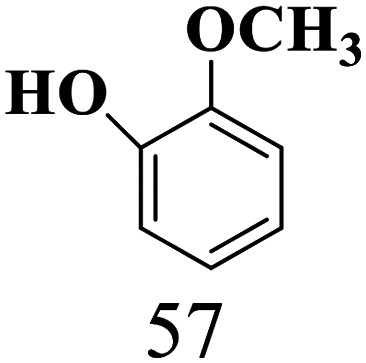
3	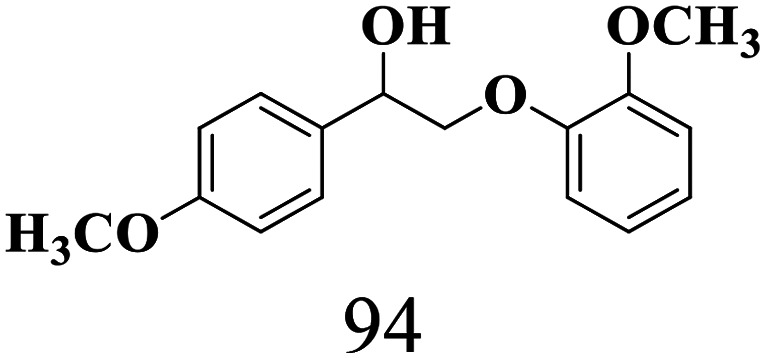	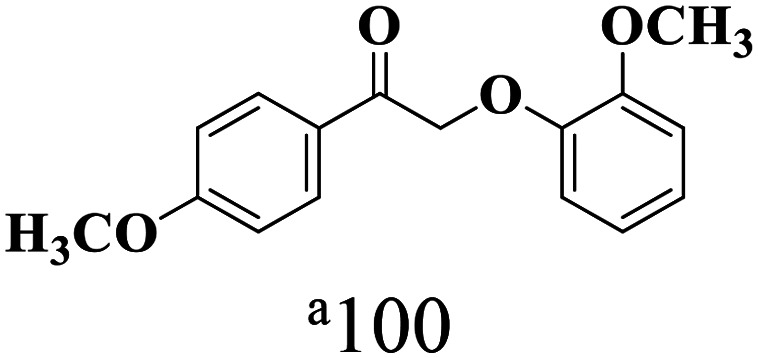	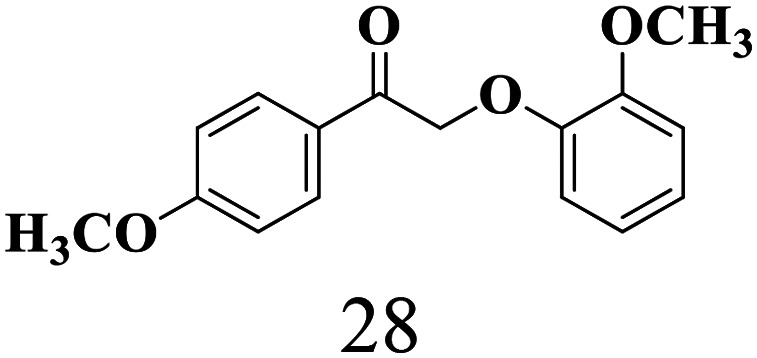	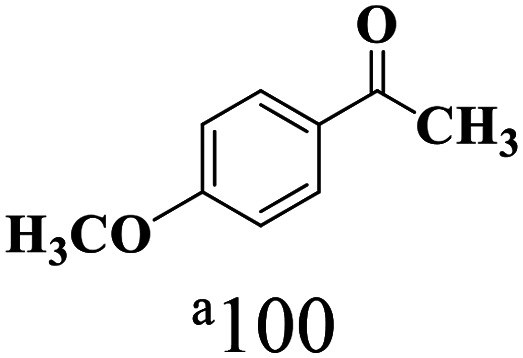	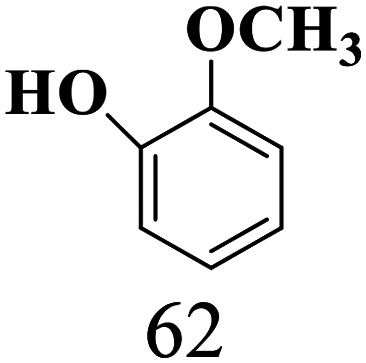

a The selectivity exceeds 100%. This inaccuracy in the selectivity value may be related to solvent evaporation or an artifact. This has been verified by GC MS analysis (Fig. S21–S25 and S35–S36†).

Within the last decade, significant advances have been made towards the development of strategies for the conversion of lignin model compounds to value-added products. However, there are still major challenges, for instance, the design of low-cost visible light absorbing catalysts based on Earth abundant transition metals to carry out the cleavage of lignin model compounds under milder and more selective conditions. Our work overcomes these challenges by LMCT-sensitization of titania by triethylammonium salts that can also act as proton donors in the reductive cleavage of β-O-4 ketones. This provides a route for the reductive cleavage of β-O-4 ketones under blue light (up to 467 nm). Beyond that, our approach utilizes the substrate (β-O-4 lignin alcohols) for LMCT-sensitization of titania in the first step that enables the oxidation of a β-O-4 lignin alcohol to a β-O-4 ketone under green light without the use of an additional oxidant by using dissolved oxygen in the reaction medium. This green and economical method allows for the transformation of lignin model compounds in one pot without any comparatively expensive photocatalysts (noble metal or rare transition metals) at ambient temperature under visible light. The mild nature of reaction conditions allows high selectivity (up to 100%) of the target fragmentation products. Hence, our approach addresses the challenges of sustainable catalysis and mild reaction conditions in lignin valorization by avoiding toxic solvents, costly photocatalysts and UV light. Developing economical and environmentally benign methods for lignin valorization is necessary for the transition toward a more sustainable economy.

## Conclusion

We successfully developed a two-step one-pot method for the cleavage of β-O-4 lignin model compounds under visible light *via* a LMCT-sensitization approach using TiO_2_ as the photocatalyst. In the first step, the β-O-4 alcohol substrate itself functioned as a sensitizer and was selectively oxidized to a β-O-4 ketone under green light using air as the oxidant. DRS-UV visible measurements show that the surface-complex formation of a β-O-4 alcohol on titania significantly improves the visible light absorption of titania (up to 700 nm). It was found that the OH group interaction of titania and β-O-4 alcohol is vital for LMCT-complex formation and subsequent selective oxidation of β-O-4 alcohol under green light. The reductive cleavage of the β-O-4 ketone was achieved under blue light, and EPR studies provide evidence that the LMCT-sensitization of titania by triethylammonium salts generates Ti^3+^ upon excitation with light, which promotes electron transfer to the substrate. Our work demonstrates that the LMCT-sensitization of titania realized the cleavage of the β-O-4 lignin model compound in one pot with a single catalyst and solvent. Additionally, the visible light-driven LMCT-sensitization of titania helped to achieve similar activity for β-O-4 ketone conversion compared to other previously reported titania-based photocatalysts under UV light (365 nm)^12,46^ and iridium-catalyzed systems under blue light.^24^ Achieving high conversion of β-O-4 ketones in one-pot reactions and scalability of these reactions remain challenges to be addressed. Overall, the outcomes of this study inspire further development in visible light-driven LMCT-mediated photocatalysis for biomass valorization and red shift of TiO_2_ photocatalysis more broadly.

## Conflicts of interest

The authors declare no conflicts of interest.

## Supplementary Material

GC-027-D5GC00948K-s001

## Data Availability

The data supporting this article have been included as part of the ESI†.
