# Supplementary material for: Visible light-driven ligand-to-metal charge transfer-mediated selective cleavage of β-O-4 lignin model compounds: a greener route to lignin valorization
Source: Green Chem. 2025 Mar 12;27(17):4664–78. doi: 10.1039/d5gc00948k (PMC11969209; doi:10.1039/d5gc00948k)
Supplement: GC-027-D5GC00948K-s001 [file GC-027-D5GC00948K-s001.pdf]

Supplementary Information (SI) for Green Chemistry.
This journal is © The Royal Society of Chemistry 2025

Supplementary Information

Visible Light-Driven Ligand-to-Metal Charge Transfer-Mediated Selective Cleavage of β-O-4 Lignin Model Compounds: A Greener Route to Lignin Valorization

Ayesha Khan, Logan W. Evans and David B.C. Martin

Department of Chemistry, University of Iowa, Iowa City, Iowa 52242, United States

Synthesis of β-O-4 lignin model compounds

All reactions were carried using oven dried or flame dried glassware charged with a magnetic stir bar and conducted under an inert nitrogen atmosphere using typical Schlenk techniques, unless otherwise noted. All solvents were dried by passage through columns of activated alumina or distilled and stored under nitrogen over freshly activated 4 Å sieves or otherwise freshly distilled. All starting materials were prepared according to known literature procedures or used as obtained from commercial sources, unless otherwise indicated. Reactions were monitored by thin-layer chromatography (TLC) and carried out on 0.25 mm coated commercial silica gel plates (Analtech TLC Uniplates, F254 precoated glass plates) using UV light as visualizing agent. Unless otherwise indicated, silica gel chromatography was performed using flash chromatography on P60 silica.

Synthesis of 2-(2-methoxyphenoxy)-1-phenylethanone (PPEn)

2-(2-methoxyphenoxy)-1-phenylethanone was prepared according to literature procedures.[1] To a 250-mL round bottom charged with a stir bar, guaiacol (17.88 mmol, 1.25 eq) and potassium carbonate (21.46 mmol, 1.5 eq) was added acetone (60.0 mL, 0.24 M) and 2-bromoacetophenone (14.30 mmol, 1 eq) with N_2 atmosphere protection. The reaction was refluxed for 4 hours, followed by filtration through celite and then concentrated *in vacuo*. Following filtration, the crude product was recrystallized from ethanol to afford the product, 2-(2-methoxyphenoxy)-1-phenylethanone as a white solid in 80% yield.

Other phenylethanone substrates were prepared via the same preparation procedures, substituting different starting materials.

Synthesis of 2-phenoxy-1-phenylethanol (PPE)

2-(2-methoxyphenoxy)-1-phenylethanol was prepared according to literature procedures.[2] To a solution of 2-(2methoxyphenoxy)-1-phenylethanone (10.36 mmol, 1 eq) in ethanol (0.15 M), with a N_2 atmosphere, was added $NaBH_4$ (12.43 mmol, 1.2 eq) and stirred at room temperature for 1 h. The suspension was diluted with water and acidified with several drops of 1M HCl and then extracted with EtOAc (10 mL, x3) and washed with brine (10 mL, x2). The organic phase was dried by anhydrous sodium sulfate. The crude mixture was purified via column chromatography (25% EtOAc/Hexane → 40% EtOAc/Hexane) to yield 2-(2-methoxyphenoxy)-1-phenylethanol in 70% yield.

Other phenylethanol substrates were prepared via the same preparation procedures, substituting different starting materials.

2-(2-methoxyphenoxy)-1-phenylethanone

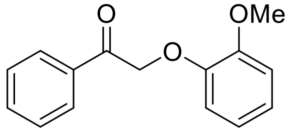

Spectral data matched literature sources.[7] ^{1}H NMR (500 MHz, $CDCl_3$) δ = 8.04-7.99 (m, 2H), 7.61 (t, J = 7.4 Hz, 1H), 7.49 (t, J = 7.4 Hz, 2H), 7.00-6.83, (m, 4H), 5.34 (s, 2H), 3.88 (s, 3H)

2-phenoxy-1-phenylethanone

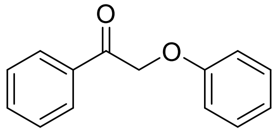

Spectral data matched literature sources.[7] Prepared from 2-bromoacetophenone and phenol in 83% yield. ^{1}H NMR (500 MHz, $CDCl_3$) δ = 8.01 (d, J= 7.4 Hz, 2H), 7.62 (t, J=7.4 Hz, 1H), 7.50 (t, J=7.7 Hz, 2H), 7.29 (t, J=8.0 Hz, 2H), 7.01-6.93 (m, 3H), 5.27 (s, 2H)

2-(2-methoxyphenoxy)-1-(4-methoxyphenyl)ethanone

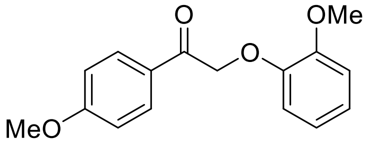

Spectral data matched literature sources.[7] Prepared from 2-bromo-4'-methoxyacetophenone and guaiacol in 82% yield. ^{1}H NMR (400 MHz, $CDCl_3$) δ = 8.02 (dd, J=6.9, 2.1 Hz, 2H), 6.99-6.89 (m, 4H), 6.86-6.82 (m, 2H), 5.28 (s, 2H), 3.89 (s, 3H), 3.88 (s, 3H)

2-(2-methoxyphenoxy)-1-phenylethanol

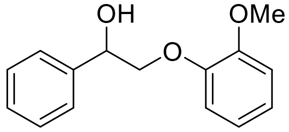

Spectral data matched literature sources.[7] Prepared from 2-(2-methoxyphenoxy)-1-phenylethanone and $NaBH_4$ in 70% yield. ^{1}H NMR (500 MHz, $CDCl_3$) δ = 7.44 (d, J=7.5 Hz, 2H), 7.37 (t, J=7.3 Hz, 2H), 7.31 (t, J=7.2 Hz, 1H), 7.02-6.88 (m, 4H), 5.11 (dd, J=9.4, 2.9 Hz, 1H), 4.20 (dd, J=10.0, 2.9 Hz, 1H), 3.98 (t, J=9.9 Hz, 1H), 3.89 (s, 3H), 2.68 (bs, 1H)

2-(2-Methoxyphenoxy)-1-(4-methoxyphenyl)ethanol

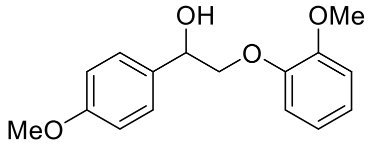

Spectral data matched literature sources.[7] Prepared from 2-(2-methoxyphenoxy)-1-(4_methoxyphenyl)ethenone and $NaBH_4$ in 91% yield. ^{1}H NMR (400 MHz, $CDCl_3$) δ = 7.36 (d, *J*=8.6 Hz, 2H), 7.02-6.87 (m, 6H), 5.05 (dd, *J*=9.3, 2.8 Hz, 1H), 4.15 (dd, *J*=10.1, 2.9 Hz, 1H), 3.96 (t, *J*=9.7 Hz, 1H), 3.89 (s, 3H), 3.81 (s, 3H).

Preparation of PPE-adsorbed titania samples

For DRS UV Visible measurements, PPE-adsorbed-SGHT-200 sample was prepared by suspending 110 mg of SGHT-200 in 37 mL of 0.5 mM PPE solution in acetonitrile. The suspension was stirred for 1 h in dark at 400 rpm. The catalyst was then collected, dried at 80 °C for 12 hours. PPE-adsorbed-P25 sample was prepared following the same method using commercial titania P25 as a catalyst. For IR measurements, the samples were prepared using 10 mM PPE solution instead of 0.5 mM solution.

Synthesis of triethylammonium salts

Triethylammonium hexafluorophosphate (TEAH PF_6) was synthesized by following a method reported by Cossairt and co-workers[3], 1g of sodium hexafluorophoshate and 1g of triethylammonium chloride were dissolved in 1 mL of water separately. The two solutions were combined, stirred thoroughly, and crystalized in a refrigerator. The resulting white crystals were collected by filtration redissolved in a minimum amount of water. TEAH PF_6 was recrystallized once again in a refrigerator and collected again by filtration. Then TEAH PF_6 was put in a scintillation vial, heated to 75 °C in oil bath under vacuum for 24 hours to remove residual water. Triethylammonium (TEAH BPh_4) was prepared in a similar way using sodium tetraphenylborate instead of sodium hexafluorophoshate.

Preparation of triethylamine-adsorbed SGHT-200

For DRS UV Visible measurements, triethylamine-adsorbed SGHT-200 (TEAH BPh_4 -SGHT-200) was prepared by adding 150 mg of SGHT-200 in 0.5 mM TEAH BPh_4 solution (15 mL) in acetonitrile in the scintillation vial. The suspension was stirred for 1 hour in dark at 400 rpm. Afterwards, the catalyst was collected, dried at 80°C for 12 hours. TEAH PF_6-SGHT-200 sample was prepared following the same method using TEAH PF_6 as triethylammonium source. For IR measurements the triethylamine-adsorbed SGHT-200 samples were prepared in a similar way, except that 10 mM triethylammonium salt solution was used.

Preparation of fluorinated titania

The surface fluorination of SGHT-200 has been performed by adding 150 mg of SGHT-200 in 15 ml of 0.053 M NaF solution. The pH of the NaF solution was adjusted to 3 using 2 M HCL. The

suspension was stirred in dark for 5 hours in dark to achieve surface fluorination. The fluorinated SGHT-200 (F-SGHT-200) was filtered and dried at 110 °C for 24 hours.

To identify the side products produced in the photocatalytic reductive cleavage of β-O-4 ketone, the following control experiments have been carried out.

Photolysis of TEAH BPh_4

Photolysis of TEAH BPh_4 has been carried out under the same conditions used for the reductive cleavage of β-O-4 ketone. The photoreactor is charged with 15 mL of 5mM TEAH BPh_4 solution in acetonitrile. The solution is irradiated with blue light (440 nm) under N_2 flow for 6 hours. The aliquots were collected and filtered using nylon syringe filters (0.22 µm) and analyzed through GC MS analysis.

Photocatalytic degradation of TEAH BPh_4

Photocatalytic degradation of TEAH BPh_4 has been studied using SGHT-200. The photoreactor is charged with 15 mL of 5mM TEAH BPh_4 solution in acetonitrile and 45 mg of SGHT-200. The suspension was stirred in dark for 1 hour and then irradiated with blue light (440 nm) under N_2 flow. The aliquots were collected and filtered using nylon syringe filters (0.22 µm) to remove the catalyst and analyzed through GC MS analysis.

Chemical stability of septum

It has been speculated that some of the compounds detected in the reaction medium may relate to the contamination from the septum that is used to cover the photoreactor. To test this hypothesis the septum was soaked in 15 mL acetonitrile overnight. Then, the aliquots were collected and filtered using nylon syringe filters (0.22 µm) and analyzed through GC MS analysis.

Quantum yield measurement

The quantum yield (Φ) is defined as the ratio of moles of product produced to the number of moles of photon absorbed by the system per unit time (Equation 1).

$$\Phi = \frac{Number\ of\ moles\ of\ product\ produced\ per\ unit\ time}{Number\ of\ moles\ of\ photons\ (Einstein)\ absorbed\ per\ unit\ time} \times 100 \qquad Equation\ 1$$

To measure the quantum yield (Φ), the photon flux to the photoreactor was determined by potassium ferrioxalate actinometry.[4,5] The actinometry experiments were carried out in a dark room under green light (515 nm) and blue light (440 nm). In a typical procedure, 15 mL of 0.15 M potassium ferrioxalate solution was charged into a glass scintillation vial used for the photocatalytic experiments. Then the actinometer solution was illuminated for 60 s while stirring (400 rpm). At the same time, another sample prepared following the same procedure was left in the dark as a control. Then, an aliquot (3 mL) of 0.15 M potassium ferrioxalate was taken in glass vials and then 0.1% buffered 1,10-phenanthroline solution (500 µL) was added to irradiated and non-irradiated samples. The samples were then allowed to develop for half an hour in the dark. After that, the absorption of each of the sample was recorded Agilent Cary 5000 UV/vis/NIR

spectrometer at 510 nm. The amount of Fe^{2+} formed during irradiation has been measured using the optical difference (ΔA 510 nm) between the irradiated and non-irradiated sample and the extinction coefficient at 510 nm ($\varepsilon = 11{,}100\ M^{-1}cm^{-1}$)

$$Nhv = \frac{moles\ of\ Fe^{2+}}{\emptyset \times t \times F} \qquad Equation\ 2$$

$$F = 1 - 10^{-A} \qquad Equation\ 3$$

The quantum yield for Fe^{2+} production is known, the photon flux absorbed by the sample per unit time is estimated using the Equation 2. Where t is the irradiation time in seconds and F is the fraction of light absorbed determined by using Equation 3.[6]

The photon flux of the photocatalytic set-up was calculated to be 14.83×10^{-9} einsteins s^{-1} and 7.85×10^{-9} einsteins s^{-1}using PR160L Kessil 525 nm lamp and PR160L Kessil 440 nm lamp, respectively.

Characterization

^{1}H and ^{13}C NMR spectra were recorded on a Bruker Avance NEO 400, Bruker Avance III 500, or Bruker Avance III 700 MHz spectrometer and were internally referenced to residual protio solvent signal (note: $CDCl_3$ referenced at δ 7.26 ppm for ^{1}H NMR and δ 77.16 ppm for ^{13}C NMR, respectively). Data for ^{1}H NMR are reported as follows: chemical shift (δ ppm), multiplicity (s = singlet, d = doublet, t = triplet, q = quartet, m = multiplet, app=apparent), coupling constant (Hz), and integration. Data for ^{13}C NMR are reported in terms of chemical shift and no special nomenclature is used for equivalent carbons. High-resolution mass spectrometry data were recorded on an Agilent 7250 GC/Q-TOF. BET specific surface area analysis was carried out on Quantachrome Nova 4200e, the samples were degassed at 110 °C for four hours prior to analysis.

For EPR analysis, the Bl-SGHT-200-TEAH BPh_4 sample was prepared by suspending 5 mg of SGHT-200 in 500 uL of 10 mM TEAH BPh_4 solution in acetonitrile. While Bl-SGHT-200-TEAH PF_6 sample was prepared by suspending 5 mg of SGHT-200 in 500 uL of 35mM TEAH PF_6 solution in acetonitrile. The samples were irradiated with blue light (440 nm) under N_2 flow and then EPR spectra of titania suspension were acquired at 100 K with a Bruker EMX (X-band) spectrometer with the aid of a Bruker ER4111VT variable temperature unit. 10 scans were obtained using signal averaging with a microwave frequency of 9.44 GHz at a power of 2.02 mW while scanning 500G/84 s with a time constant of 164 ms. The receiver was set with a modulation amplitude of 5.14 G with a modulation frequency of 100-kHz, and a receiver gain of 5.02×10^{5}. The high-resolution TEM images were taken via Hitachi HT-7800 transmission electron microscope using formvar/carbon supported copper grids (grid size 400 mesh) as support. The average particle size was estimated by counting 150 particles.

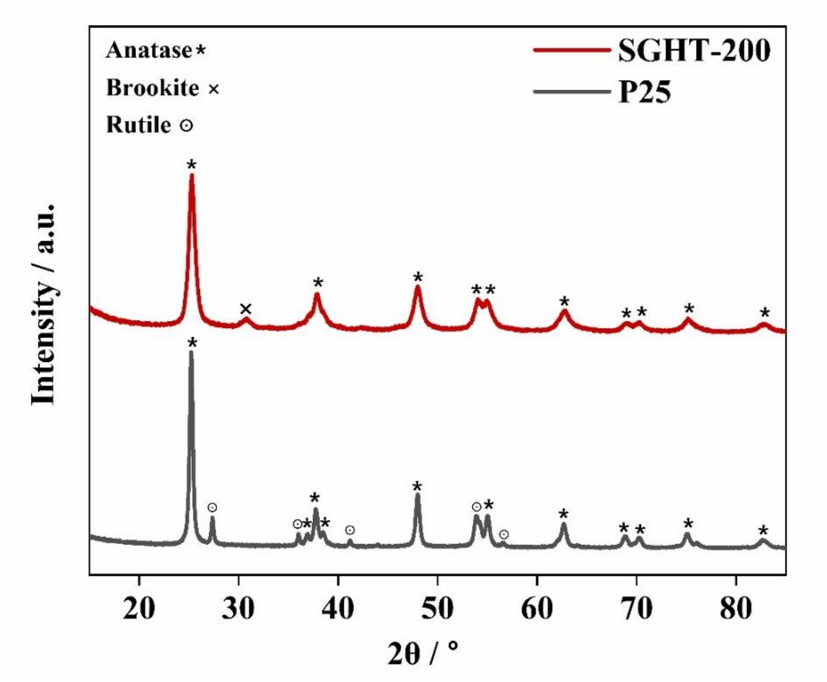

Fig. S1 X-ray diffraction (XRD) patterns of lab-synthesized titania (SGHT-200) and commercial titania (P25).

Table S1 Crystallographic features and textural properties (Brunauer-Emmett-Teller specific surface area [SSA], Barrett-Joyner-Halenda [BJH] pore volume and pore radius) of lab-synthesized titania (SGHT-200) and commercial titania (P25).

**Entry**	**Catalyst**	**Ratio of crystalline phases (%)**	**Crystal size (nm)**			**SSA (m^2g^{-1})**	**BJH pore radius (Å)**	**BJH pore volume (cm^3g^{-1})**
		**Anatase:Brookite:Rutile**	**Anatase**	**Brookite**	**Rutile**			
1	SGHT-200	79:21:0	11	8	NA	109	55	0.335
2	P25	87:0:13	25	NA	42	46	171	0.465

NA: Not applicable

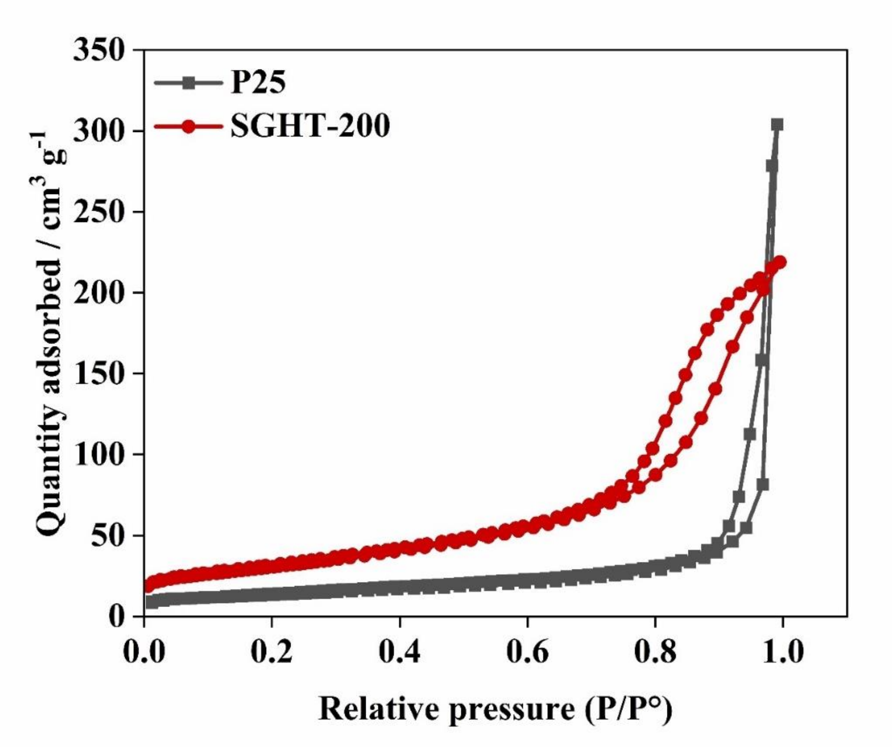

Fig. S2 Nitrogen adsorption-desorption isotherms of lab-synthesized titania (SGHT-200) and commercial titania (P25).

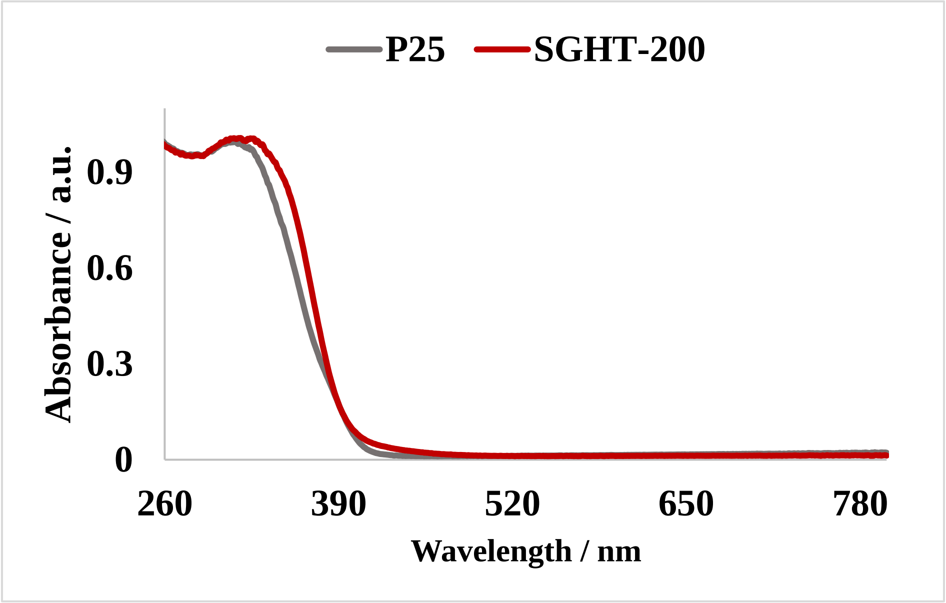

Fig. S3 DRS-UV-Visible absorption spectra of lab-synthesized titania (SGHT-200) and commercial titania (P25).

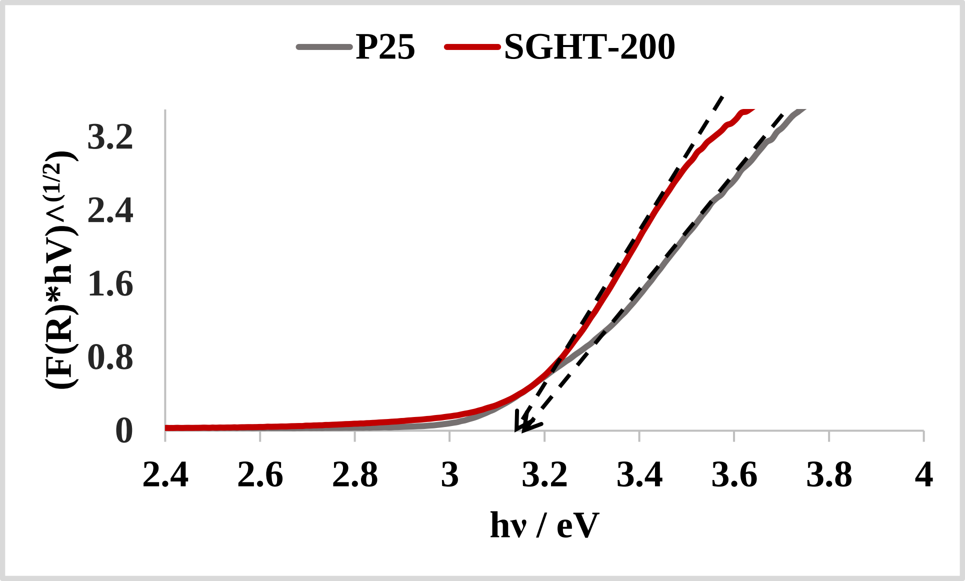

Fig. S4 Optical band gap of lab-synthesized titania (SGHT-200) and commercial titania (P25).

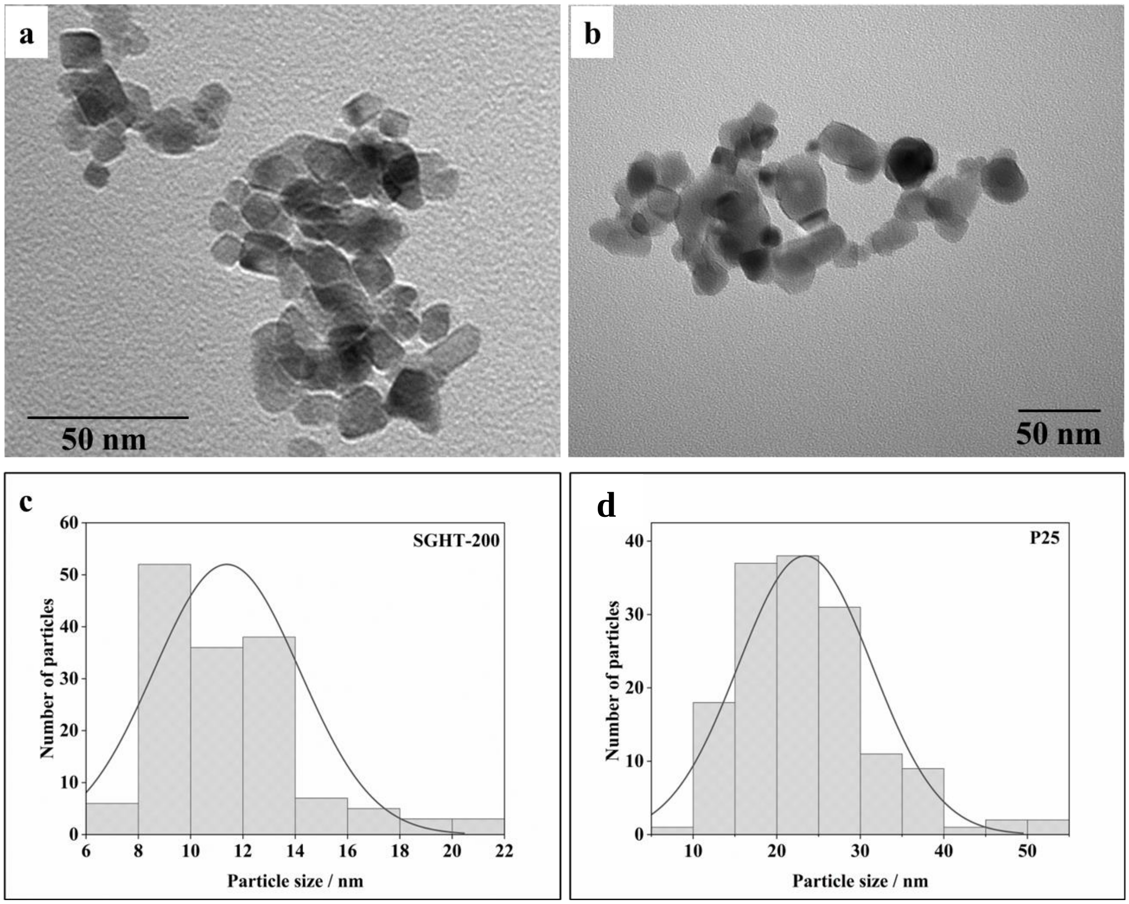

Fig. S5 High resolution TEM images of a) SGHT-200 b) P25 and particle size distribution of a) SGHT-200 b) P25

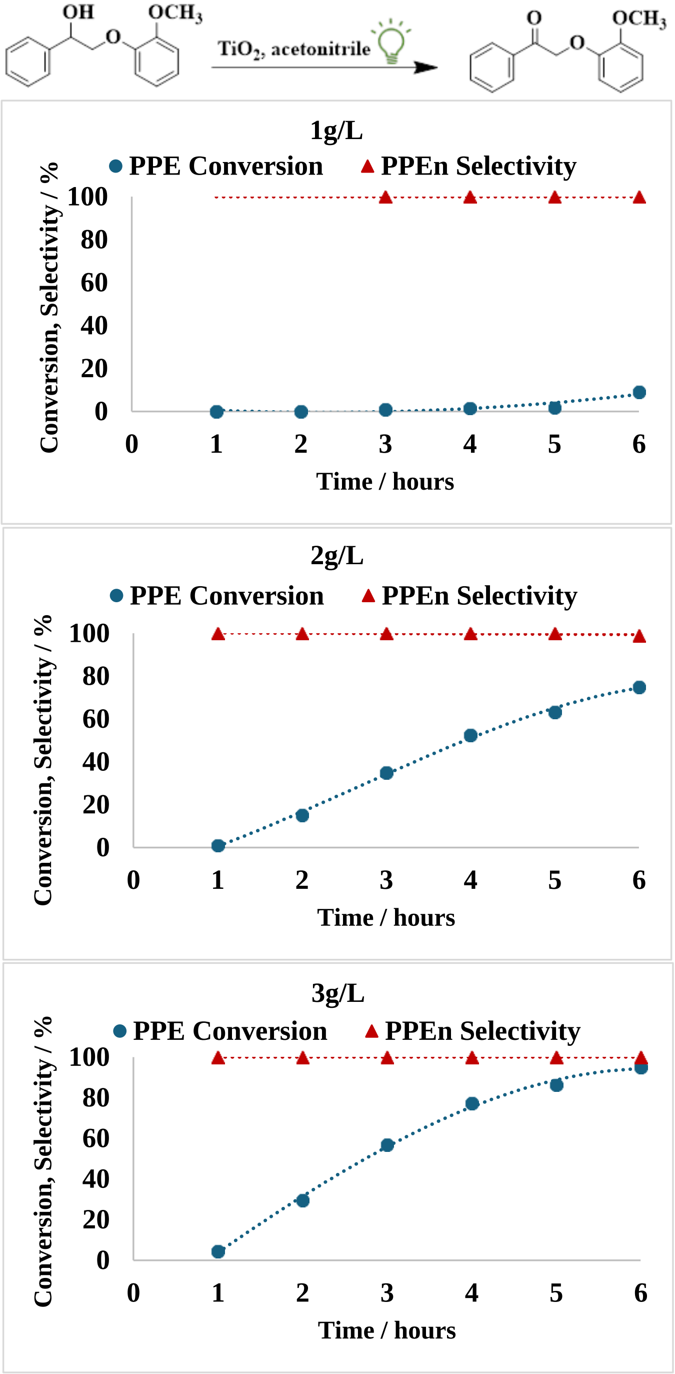

Fig. S6 Effect of catalyst loading on the photocatalytic performance of SGHT-200 in the selective oxidation of 2-(2-methoxyphenoxy)-1-phenylethanol (PPE) to 2-(2-methoxyphenoxy)-1-phenylethanone (PPEn) under green light (525 nm).

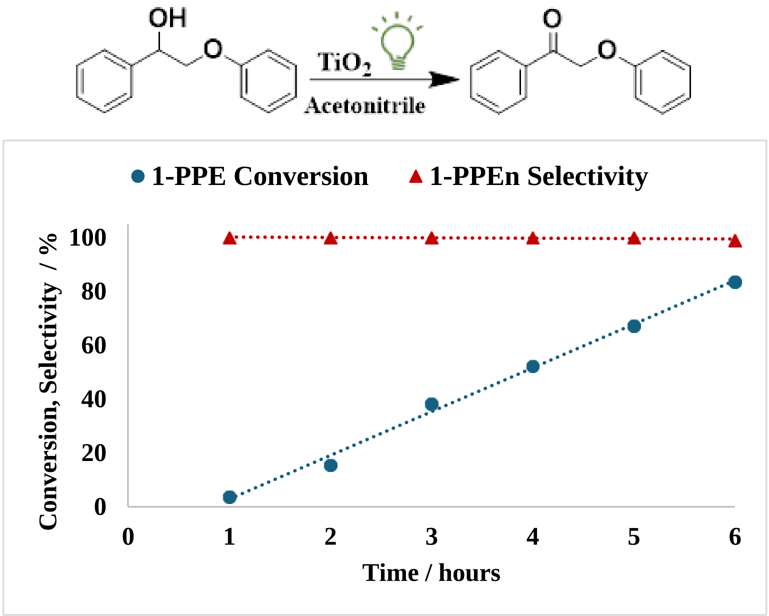

Fig. S7 Effect of reaction time on the photocatalytic performance of SGHT- 200 (3g/L) in the selective oxidation of 2-phenoxy-1-phenylethanol (1-PPE) to 2-phenoxy-1-phenylethanone (1-PPEn) under green light (525 nm)

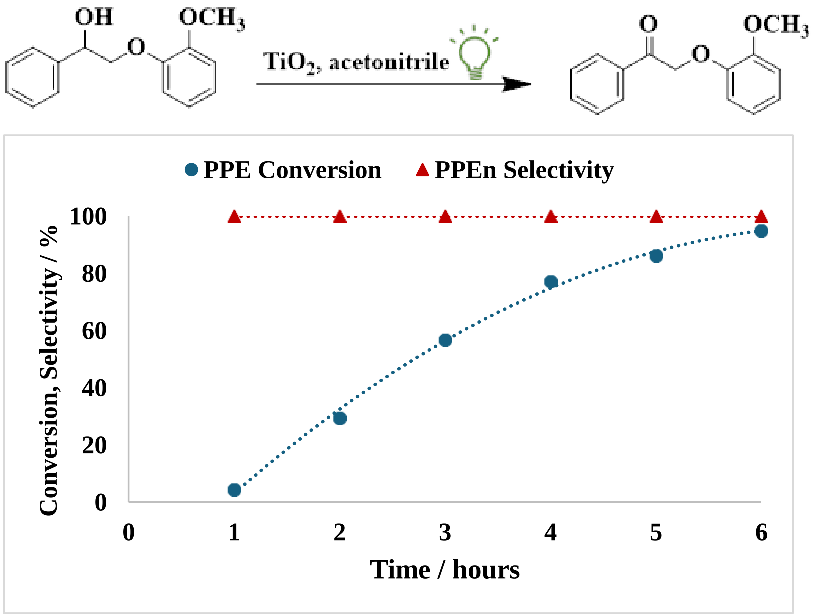

Fig. S8 Effect of reaction time on the photocatalytic performance of SGHT- 200 (3g/L) in the selective oxidation of 2-(2-methoxyphenoxy)-1-phenylethanol (PPE) to 2-(2-methoxyphenoxy)-1-phenylethanone (PPEn) under green light (525 nm).

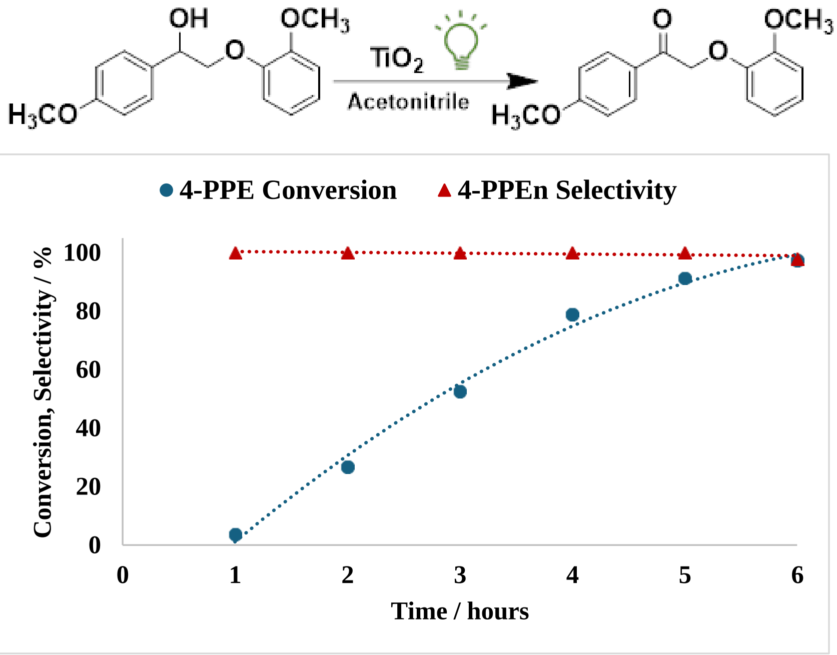

Fig. S9. **Effect of reaction time on the photocatalytic performance of SGHT- 200 (3g/L) in the selective oxidation of 2-(2-methoxyphenoxy)-1-(4-methoxyphenyl)ethanol (4-PPE) to 2-(2-methoxyphenoxy)-1-(4-methoxyphenyl)ethenone (4-PPEn) under green light (525 nm).**

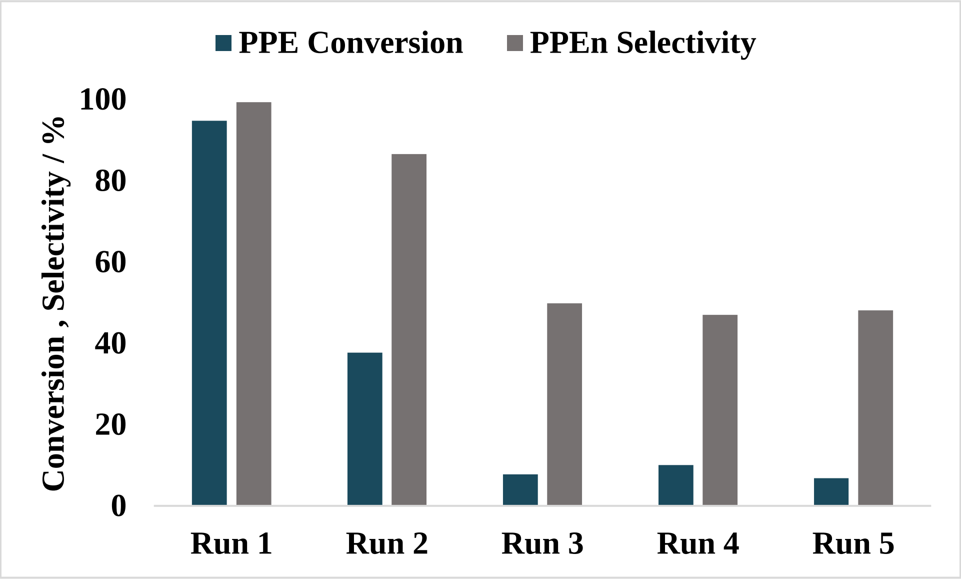

Fig. S10 Recyclability test for SGHT-200 for the selective oxidation of 2-(2-methoxyphenoxy)-1-phenylethanol (PPE) to 2-(2-methoxyphenoxy)-1-phenylethanone (PPEn) under green light (525 nm).

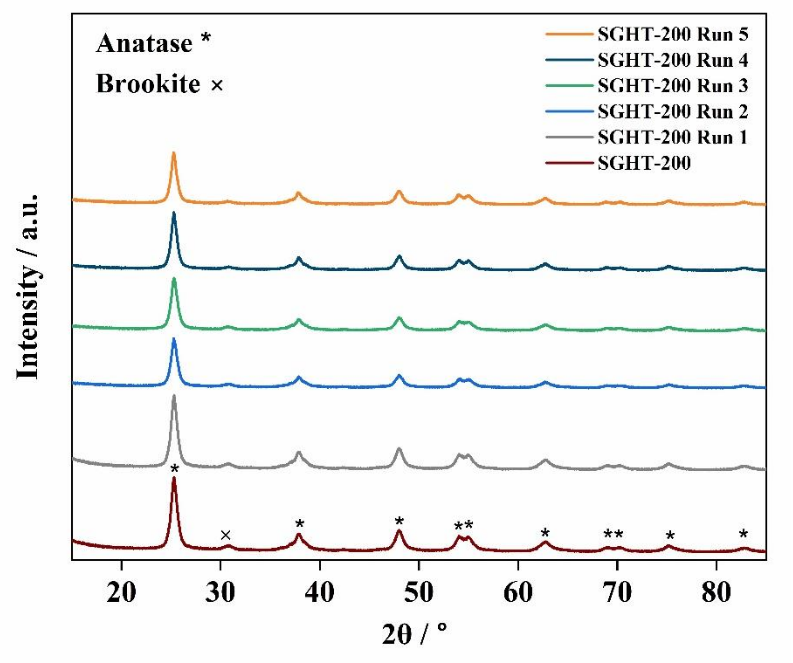

Fig. S11 X-ray diffraction (XRD) patterns of SGHT-200 reused in in selective oxidation of 2-(2-methoxyphenoxy)-1-phenylethanol (PPE) to 2-(2-methoxyphenoxy)-1-phenylethanone (PPEn) under green light (525 nm)

Table S2: Crystallographic features and textural properties (Brunauer-Emmett-Teller specific surface area [SSA], Barrett-Joyner-Halenda [BJH] pore volume and pore radius) of SGHT-200 reused in selective oxidation of 2-(2-methoxyphenoxy)-1-phenylethanol (PPE) to 2-(2-methoxyphenoxy)-1-phenylethanone (PPEn) under green light (525 nm).

Entry	Catalyst	Ratio of crystalline phases (%)	Crystal size (nm)		SSA (m^2g^{-1})	BJH pore radius (Å)	BJH pore volume (cm^3g^{-1})
		Anatase:Brookite	Anatase	Brookite			
1	SGHT-200	79:21	11	8	109	55	0.335
2	SGHT-200 Run 1	80:20	11	5	93	54	0.308
3	SGHT-200 Run 2	80:20	11	9	96	60	0.360
4	SGHT-200 Run 3	80:20	11	9	72	59	0.232
5	SGHT-200 Run 4	85:15	12	8	79	54	0.252
6	SGHT-200 Run 5	80:20	11	6	104	55	0.351

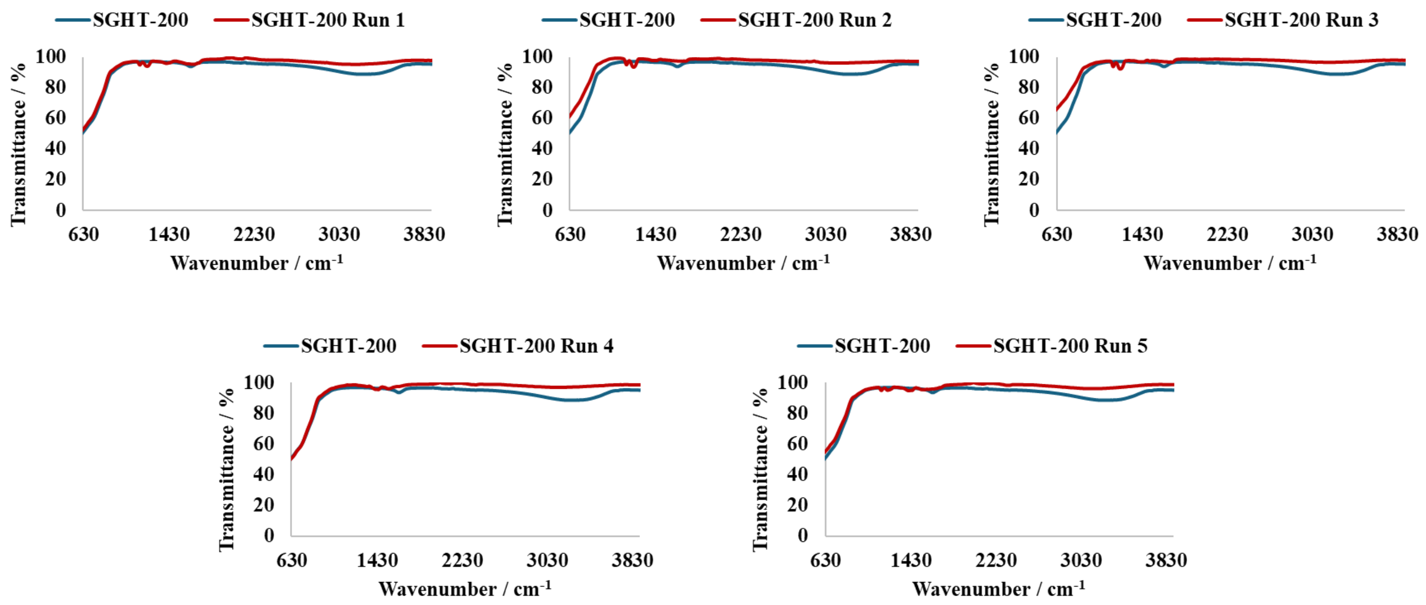

Fig. S12 IR analysis of SGHT-200 reused in selective oxidation of 2-(2-methoxyphenoxy)-1-phenylethanol (PPE) to 2-(2-methoxyphenoxy)-1-phenylethanone (PPEn) under green light (525 nm) and washed with acetonitrile.

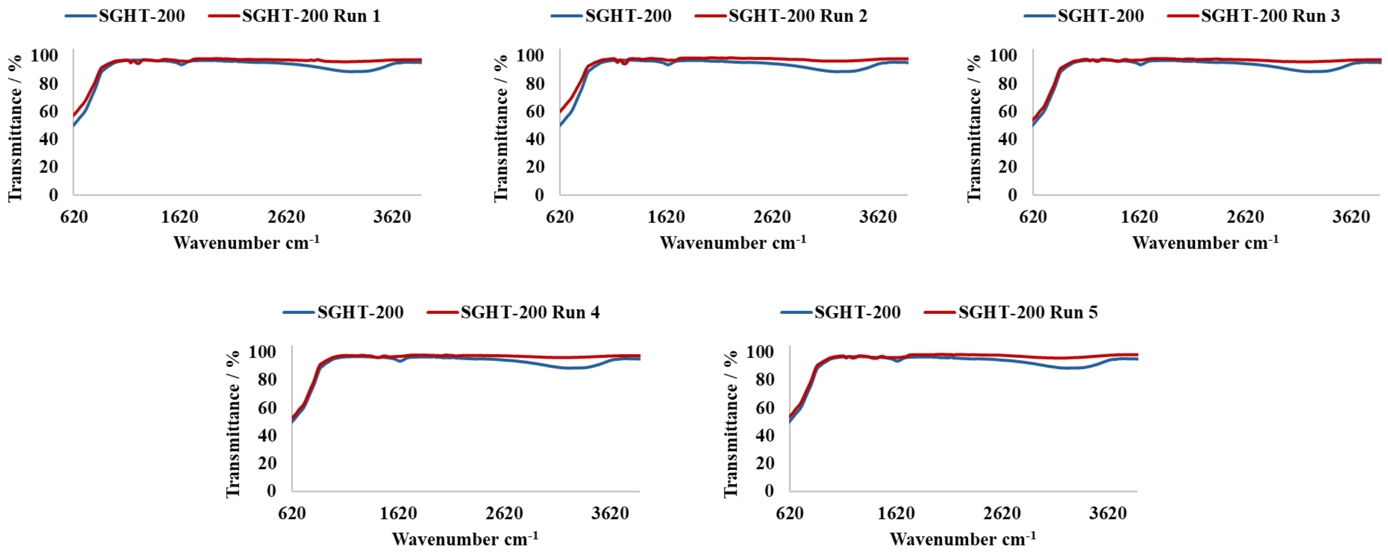

Fig. S13 IR analysis of SGHT-200 reused in selective oxidation of 2-(2-methoxyphenoxy)-1-phenylethanol (PPE) to 2-(2-methoxyphenoxy)-1-phenylethanone (PPEn) under green light (525 nm) and washed with water.

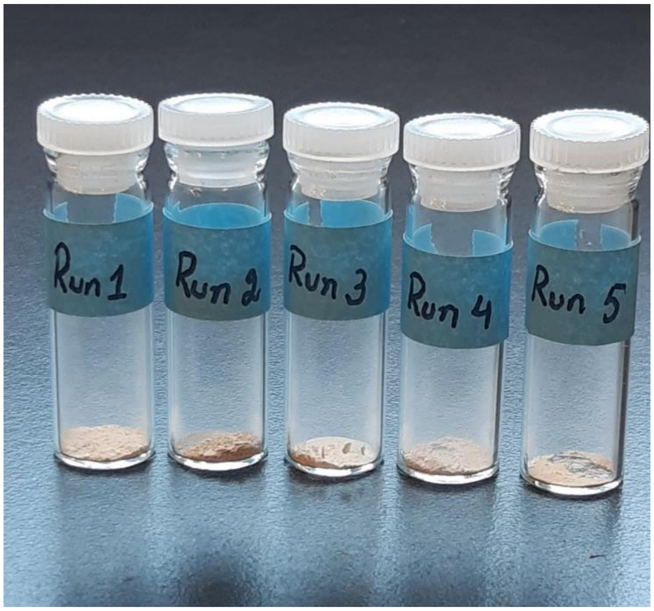

Fig. S 14 SGHT-200 after multiple runs in selective oxidation of 2-(2-methoxyphenoxy)-1-phenylethanol (PPE) to 2-(2-methoxyphenoxy)-1-phenylethanone (PPEn) under green light (525 nm).

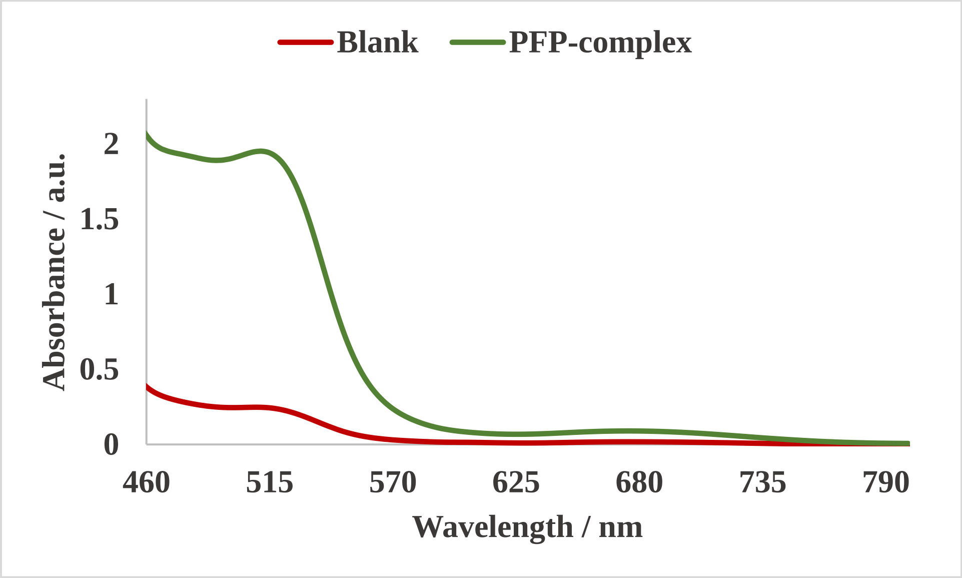

Fig. S15 UV-Visible absorption spectrum of potassium-ferrioxalate phenanthroline (PFP) complex after green light (525 nm) irradiation.

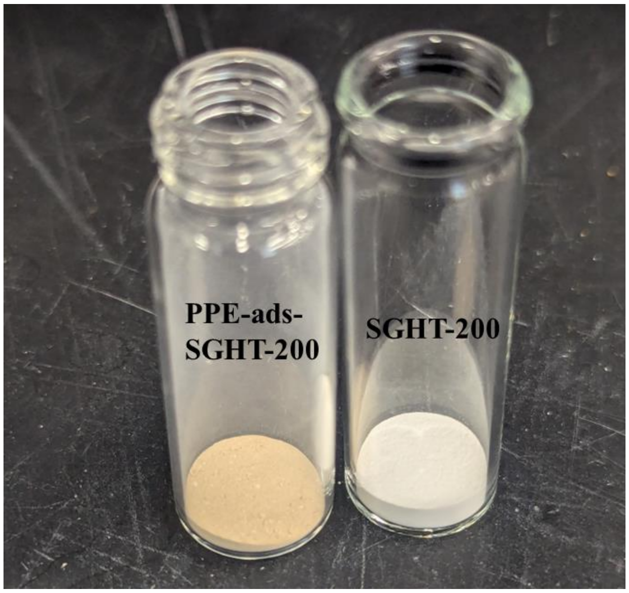

Fig. S16 SGHT-200 and 2-(2-methoxyphenoxy)-1-phenylethanol (PPE)- adsorbed SGHT-200.

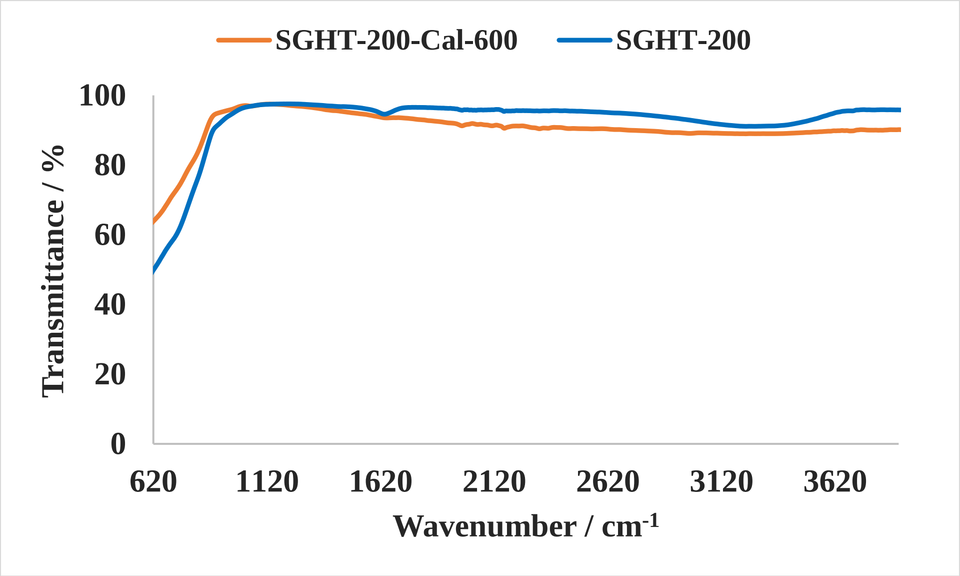

Fig. S17 IR spectra of SGHT-200 and calcined titania (SGHT-200-Cal-600).

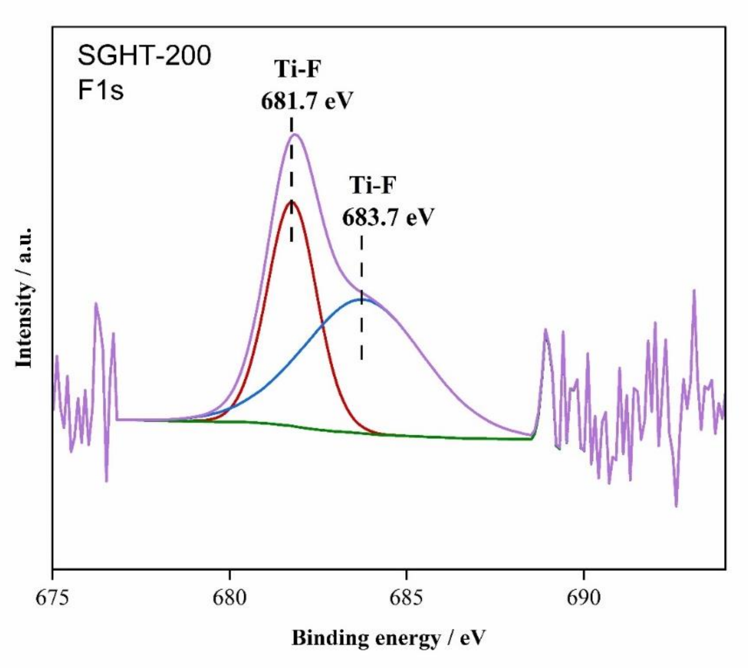

Fig. S18 F1s XPS spectra of fluorinated SGHT-200 (F-SGHT-200).

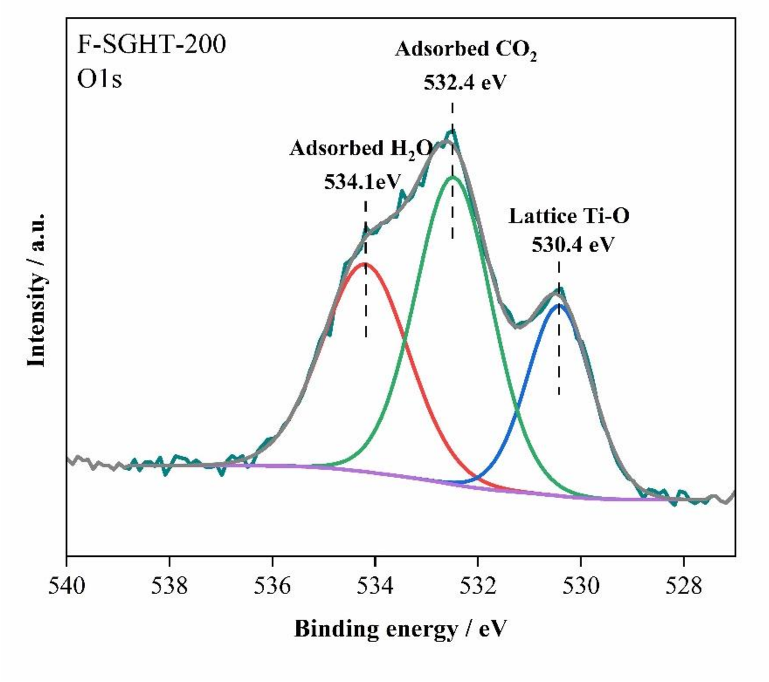

Fig. S19 O1s XPS spectra of fluorinated SGHT-200 (F-SGHT-200).

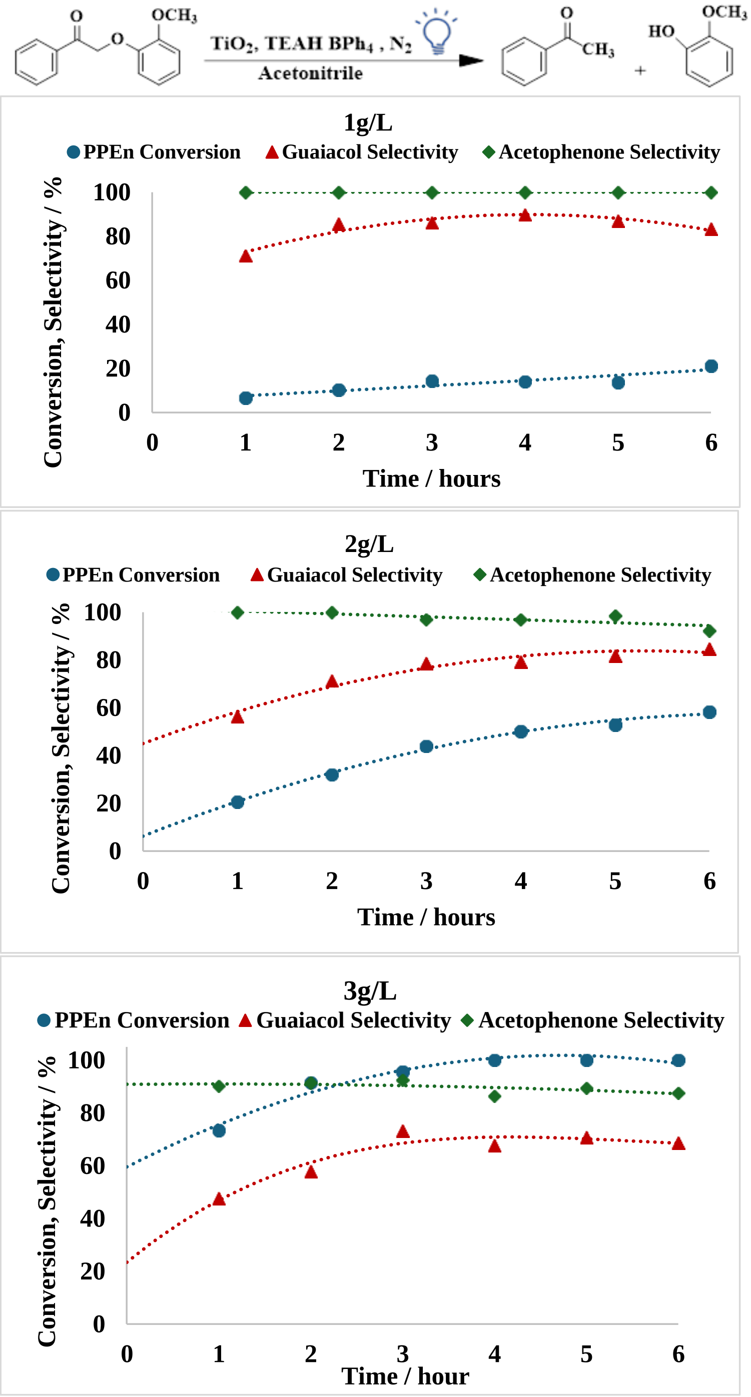

Fig. S20 Effect of catalyst loading on the photocatalytic performance of SGHT-200 in the reductive cleavage of 2-(2-methoxyphenoxy)-1-phenylethanone (PPEn) under blue light (440 nm).

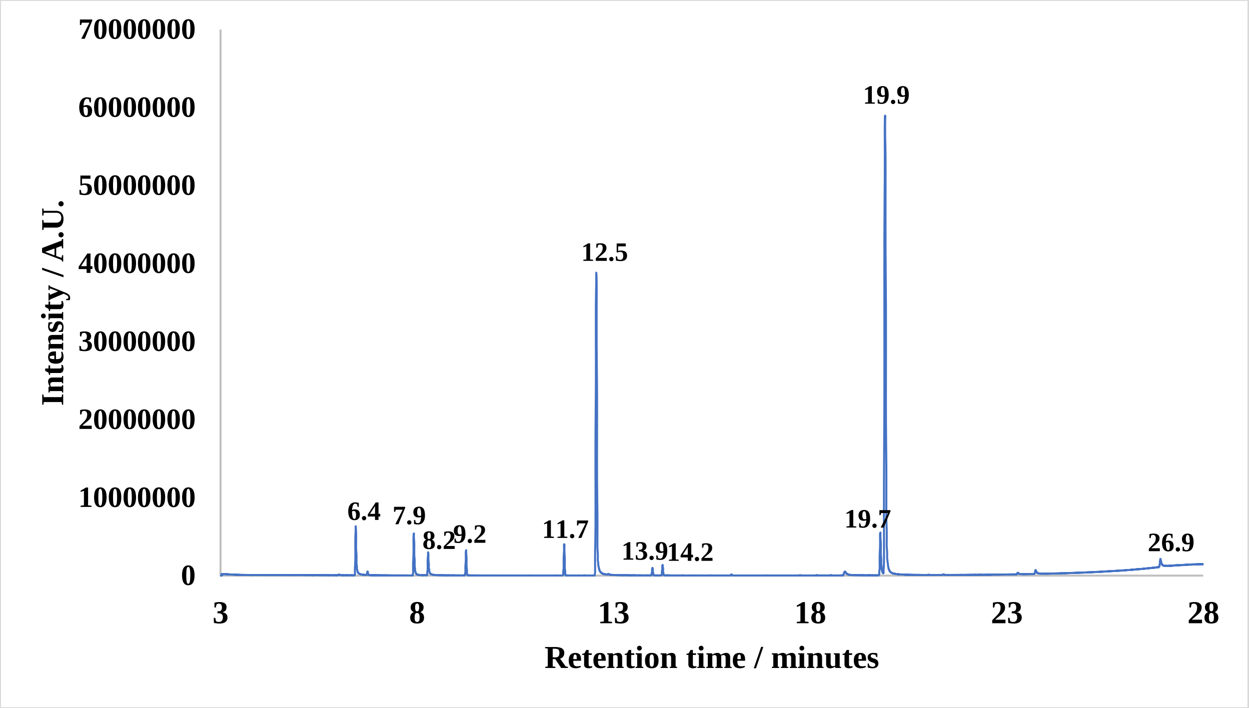

Fig. S21 GC chromatograms of photocatalytic reductive cleavage of β-O-4 ketone (PPEn) in acetonitrile in the presence of TEAH BPh_4.

The peaks detected at 6.4 minutes, 7.9 minutes, 8.2 minutes, 9.2 minutes, 11.7 minutes, 12.5 minutes, 13.9 minutes, 14.2 minutes, 18.8 minutes, 19.7 minutes, 19.9 minutes and 26.9 minutes are assigned to phenol, acetophenone, guaiacol, decamethylcyclopentasiloxane, dodecamethylcyclohexasiloxane, biphenyl, tetradecamethylcycloheptasiloxane, butylated hydroxytoluene, benzene, 2-(2-Methoxyphenoxy)-1-phenylethanone, triphenylborane and hexamethylcyclotrisiloxane, respectively.

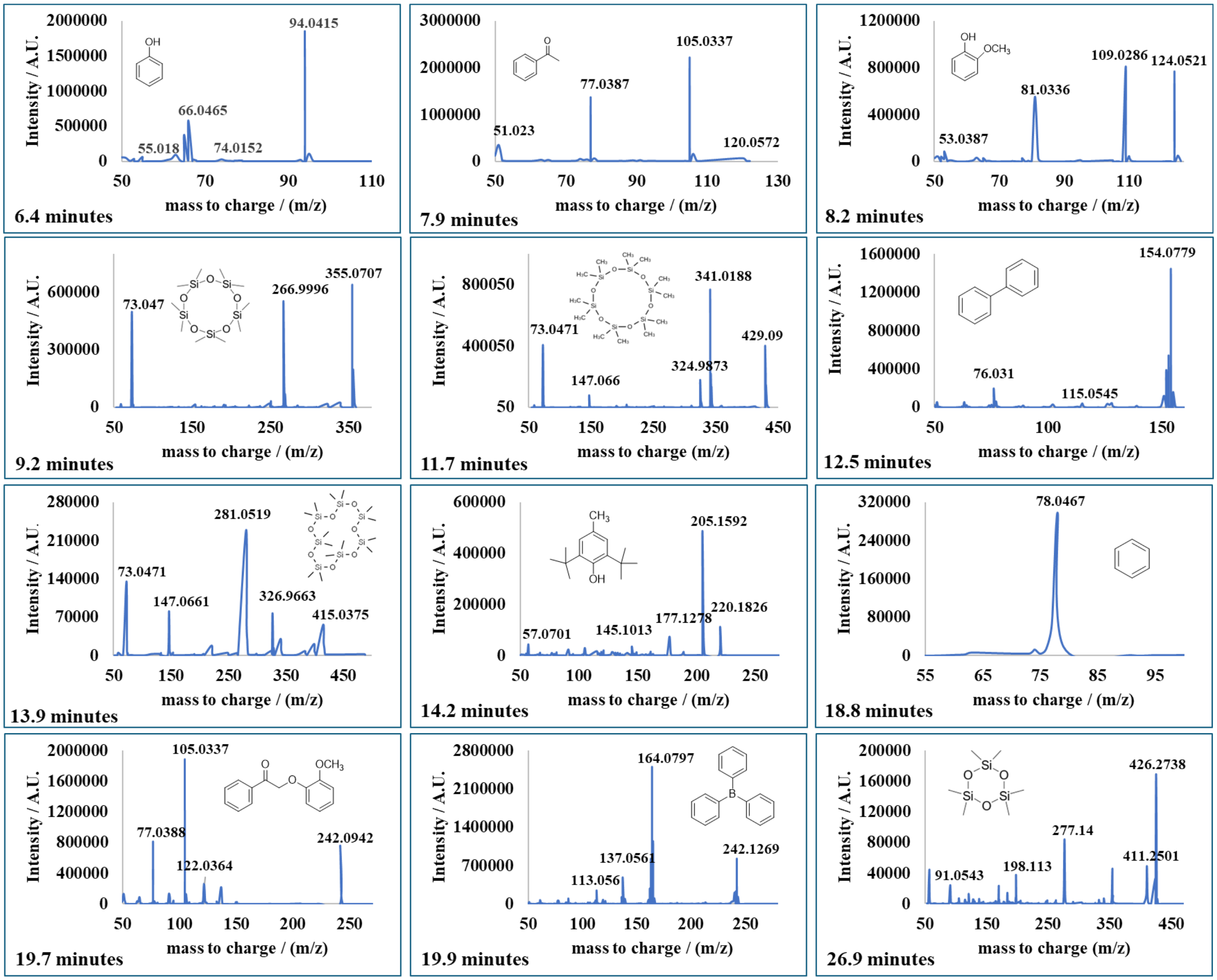

Fig. S22 Mass spectrum of compounds detected in the photocatalytic reductive cleavage of β-O-4 ketone (PPEn) in acetonitrile in the presence of TEAH BPh_4.

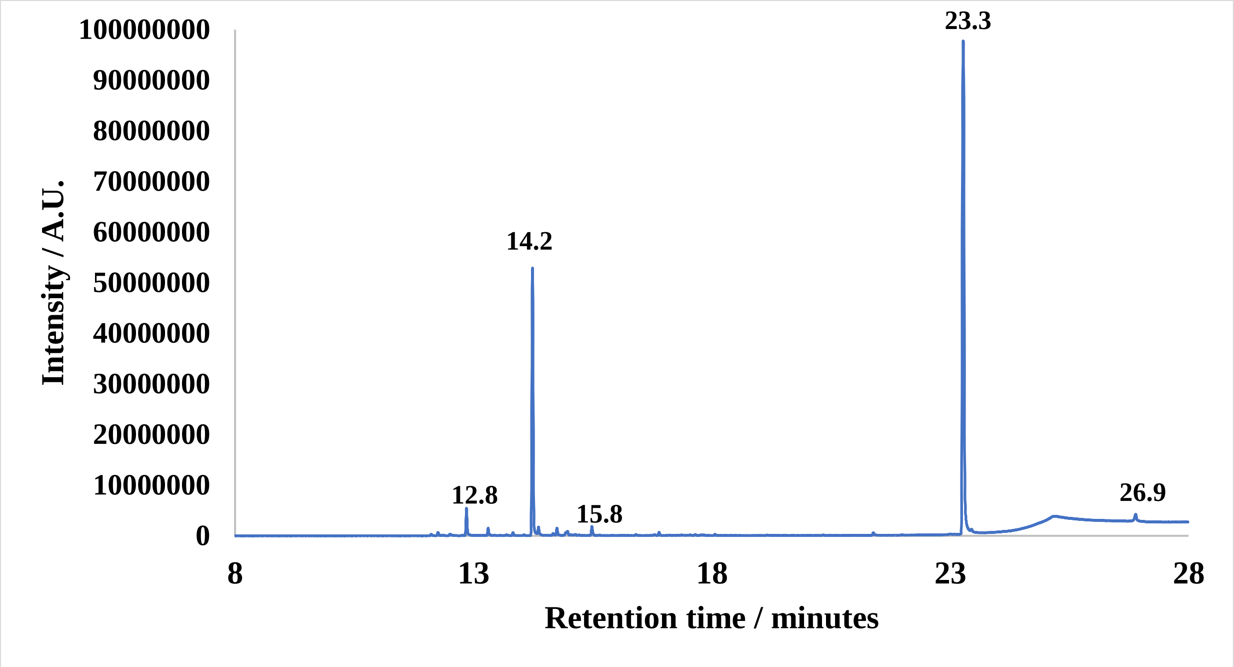

Fig. S23 GC chromatogram for contaminants leached from the septum.

The peaks detected at 12.8 minutes, 14.2 minutes, 15.4 minutes and 26.8 minutes are assigned to diphenyl ether, butylated hydroxytoluene, 2,2'-Methylenebis[4-Methyl-6-tert-butylphenol], hexamethyl cyclotrisiloxane, respectively.

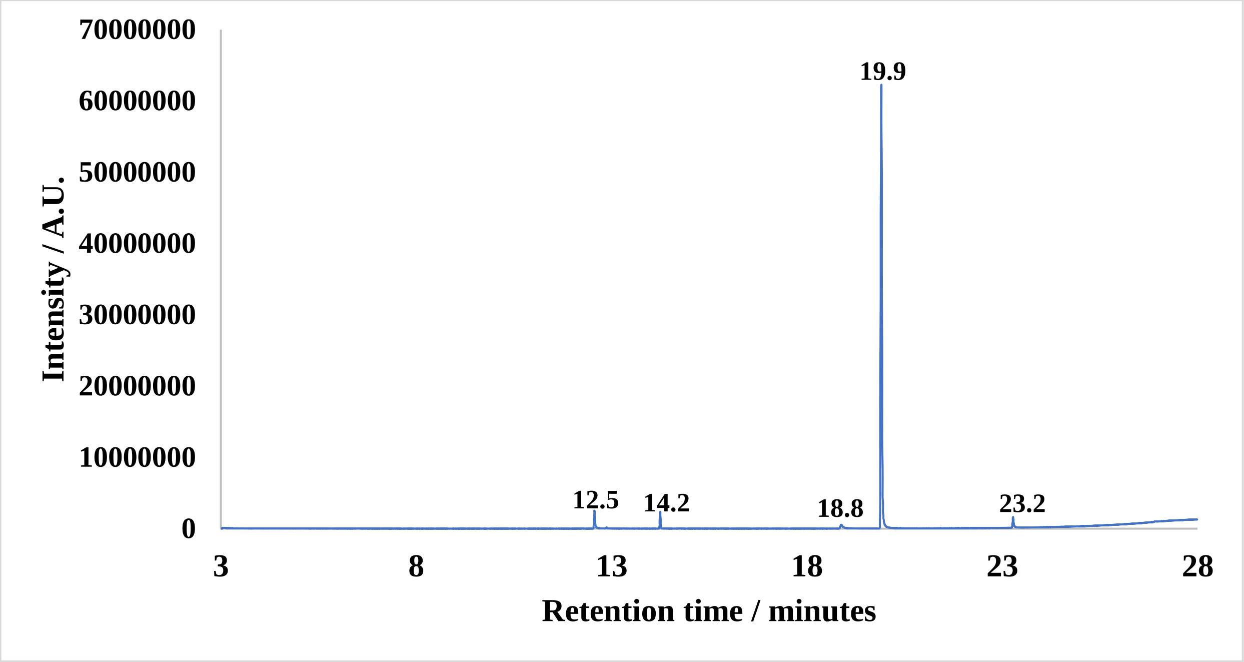

Fig. S24 GC chromatograms of products obtained through photocatalytic degradation of TEAH BPh_4 in acetonitrile.

The peaks detected at 12.5 minutes, 14.2 minutes, 18.8 minutes, 19.9 minutes and 23.2 minutes are assigned to biphenyl, butylated hydroxytoluene, benzene, triphenyl borane 2,2'-Methylenebis[4-Methyl-6-tert-butylphenol], respectively.

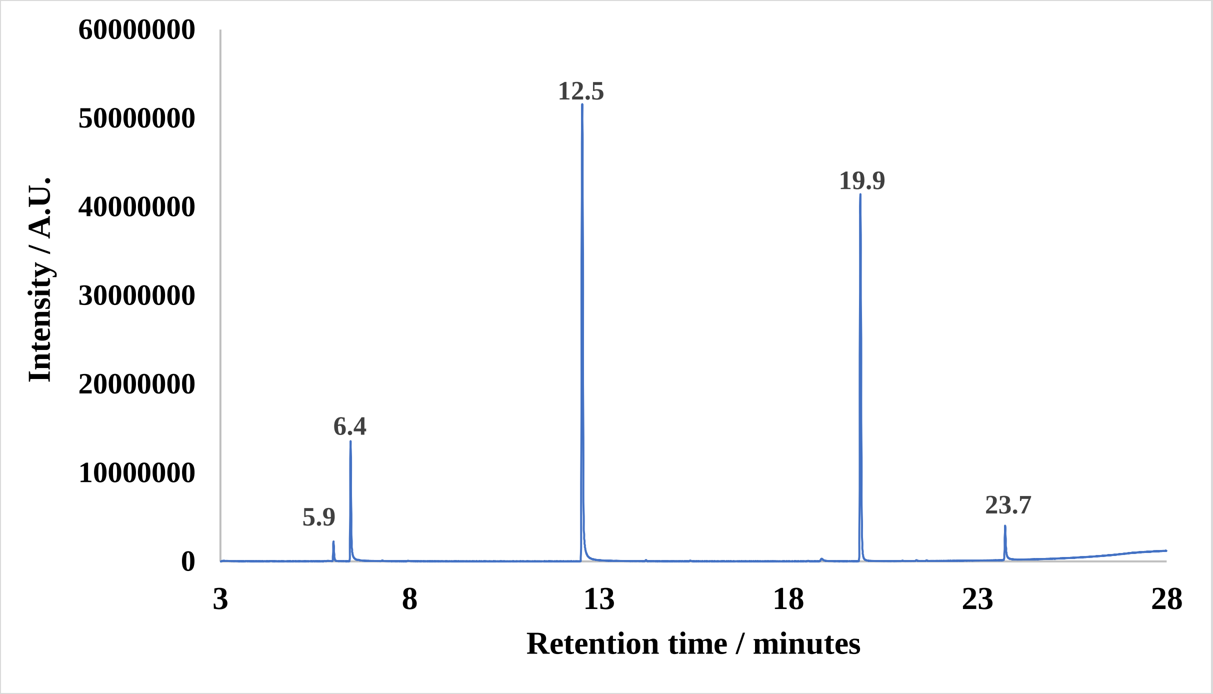

Fig. S25 GC chromatograms of products obtained through photolysis of TEAH BPh4.

The peaks detected at 5.5 minutes, 6.4 minutes, 12.5 minutes, 18.8 minutes, 19.9 minutes and 23.7 minutes are assigned to 1-isopropyl-3-methyl-2-pyrazoline, phenol, biphenyl, benzene, triphenyl borane, triphenyl boroxin, respectively.

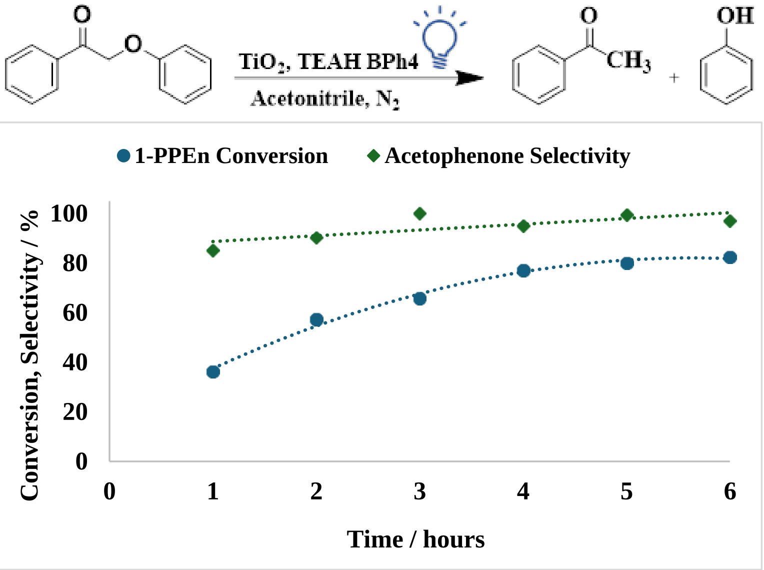

Fig. S26 Effect of reaction time on the photocatalytic performance of SGHT- 200 (3g/L) in the reductive cleavage of of 2-phenoxy-1-phenylethanone (1-PPEn) under blue light (440 nm). Note: Selectivity of phenol cannot be determined accurately as the degradation of TEAH BPh4 also produces phenol.

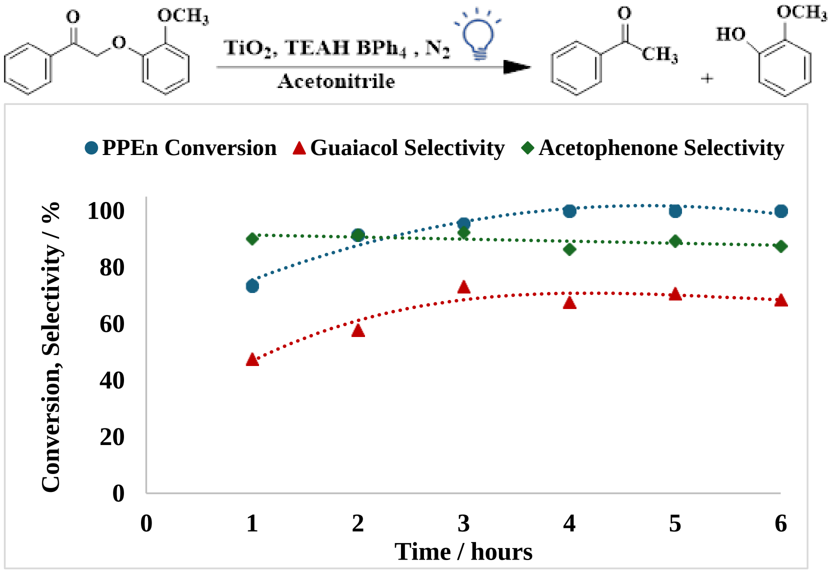

Fig. S27 Effect of reaction time on the photocatalytic performance of SGHT- 200 (3g/L) in the reductive cleavage of 2-(2-methoxyphenoxy)-1-phenylethanone (PPEn) under blue light (440nm).

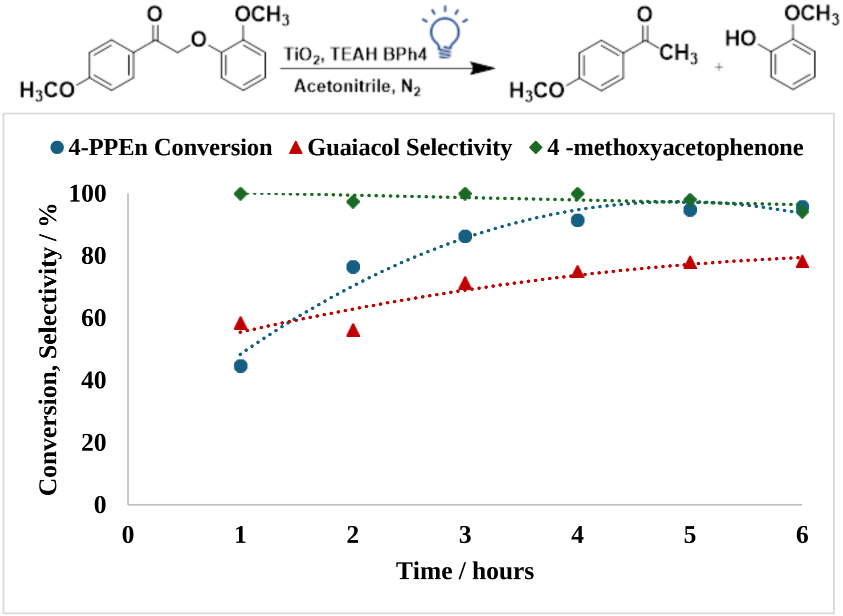

Fig. S28 Effect of reaction time on the photocatalytic performance of SGHT- 200 (3g/L) in the reductive cleavage of of 2-(2-methoxyphenoxy)-1-(4-methoxyphenyl)ethanone (4-PPEn) under blue light (440nm).

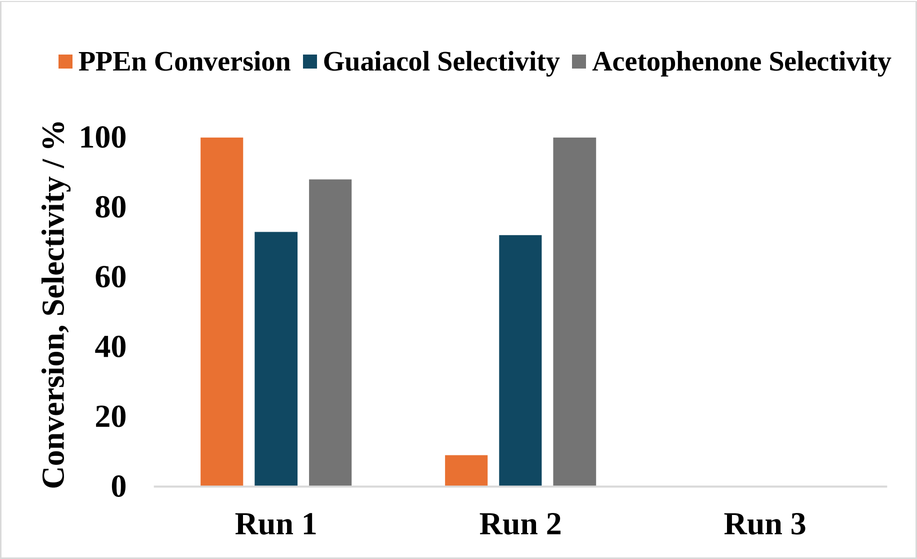

Fig. S29 Recyclability test for SGHT-200 for the reductive cleavage of 2-(2-methoxyphenoxy)-1-phenylethanone (PPEn) under blue light (440 nm).

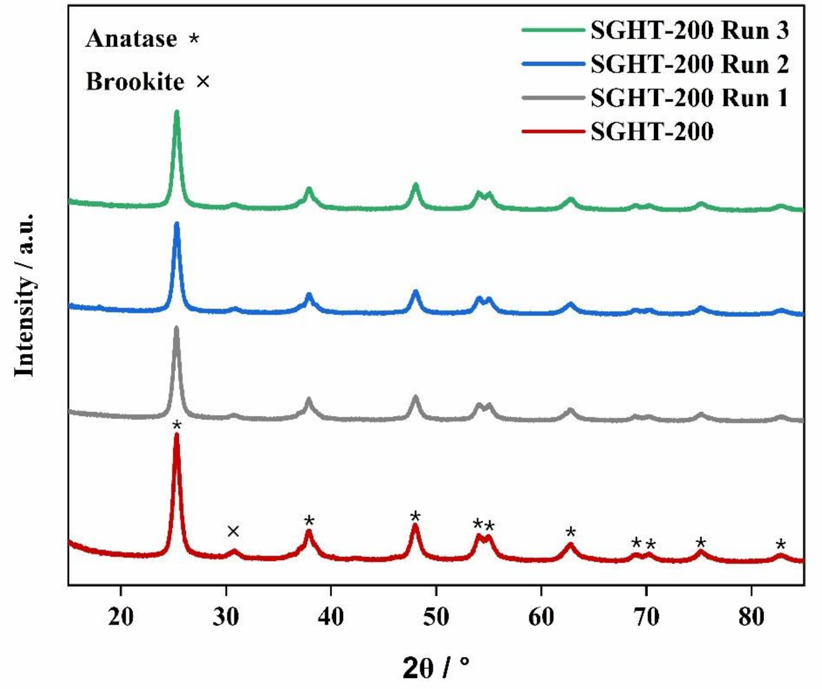

Fig. S30 X-ray diffraction (XRD) patterns of SGHT-200 after multiple runs in reductive cleavage of 2-(2-methoxyphenoxy)-1-phenylethanone (PPEn) under blue light (440 nm).

Table S3 Crystallographic features and textural properties (Brunauer-Emmett-Teller specific surface area [SSA], Barrett-Joyner-Halenda [BJH] pore volume and pore radius) of SGHT-200 reused in reductive cleavage of 2-(2-methoxyphenoxy)-1-phenylethanone (PPEn) under blue light (440 nm).

Entry	Catalyst	Ratio of crystalline phases (%)	Crystal size (nm)		SSA (m^2g^{-1})	BJH pore radius (Å)	BJH pore volume (cm^3g^{-1})
		Anatase:Brookite	Anatase	Brookite			
1	SGHT-200	79:21	11	8	109	55	0.335
2	SGHT-200 Run 1	79:21	11	6	79	49	0.254
3	SGHT-200 Run 2	85:15	12	8	76	54	0.347
4	SGHT-200 Run 3	85:15	12	8	98	54	0.358

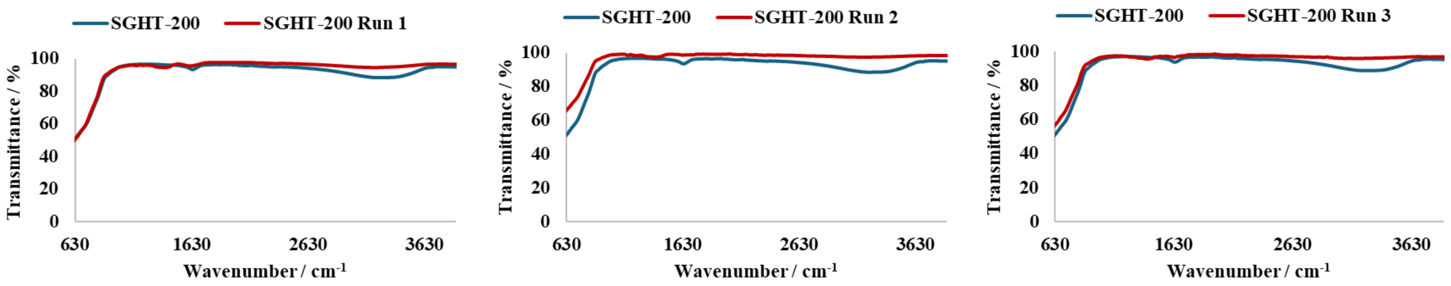

Fig. S31 IR analysis of SGHT-200 reused in in reductive cleavage of 2-(2-methoxyphenoxy)-1-phenylethanone (PPEn) under blue light (440 nm) and washed with acetonitrile.

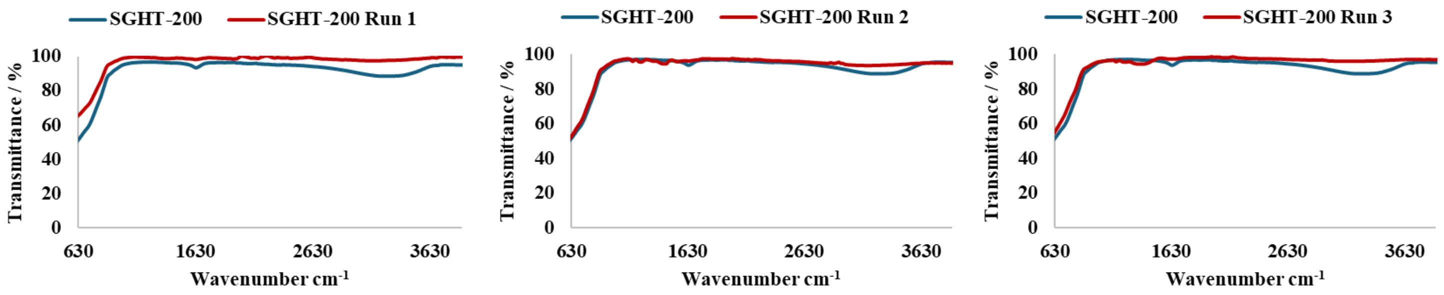

Fig. S32 IR analysis of SGHT-200 reused in reductive cleavage of 2-(2-methoxyphenoxy)-1-phenylethanone (PPEn) under blue light (440 nm) and washed with water.

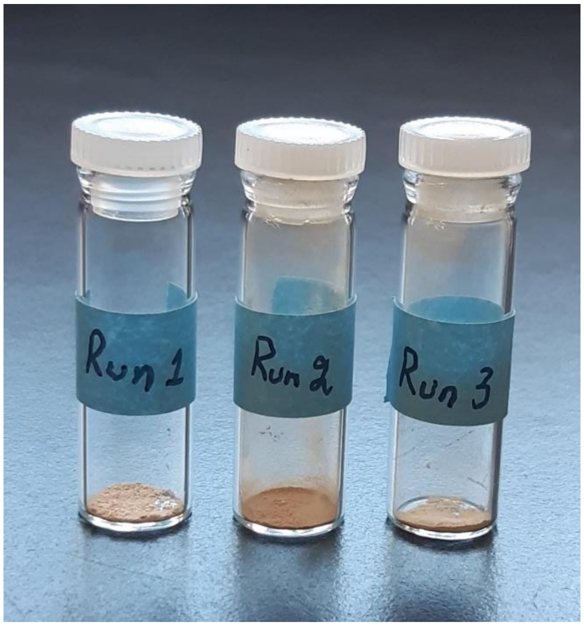

Fig. S33 SGHT-200 after multiple runs in the reductive cleavage of 2-(2-methoxyphenoxy)-1-phenylethanone (PPEn) under blue light (440 nm).

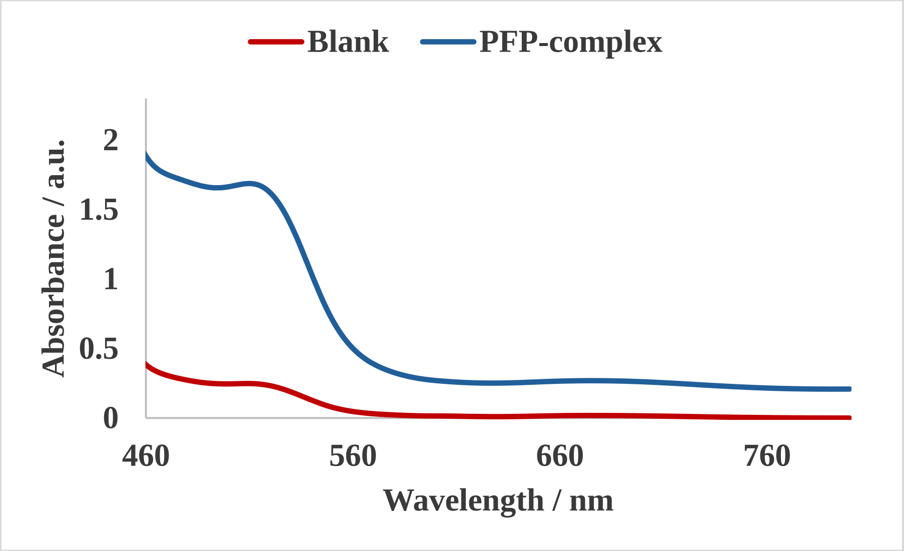

Fig. S34 UV-Visible absorption spectrum of potassium–ferrioxalate phenanthroline (PFP) complex after blue light (440 nm) irradiation.

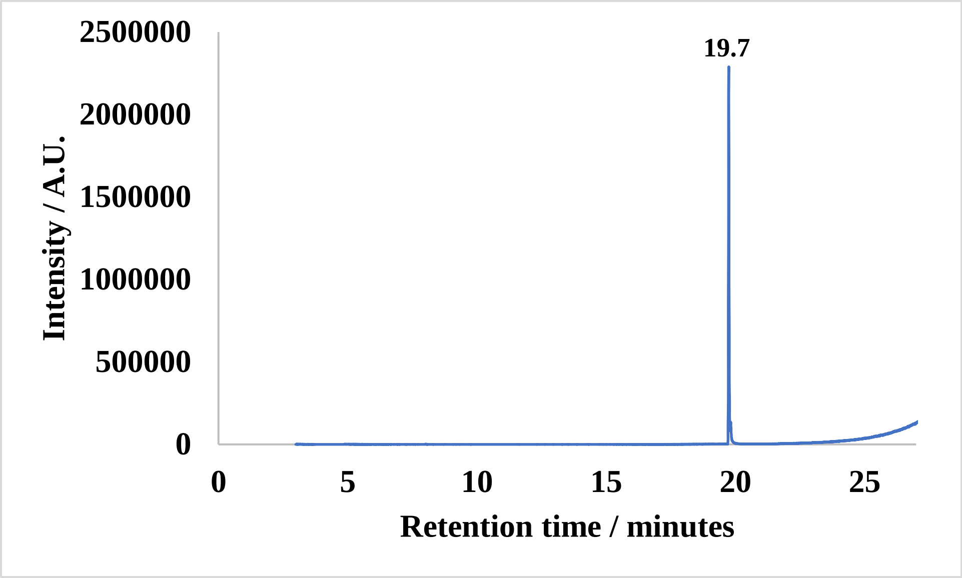

Fig. S35. GC chromatograms of photocatalytic selective oxidation of β-O-4 alcohol (PPE) product in acetonitrile.

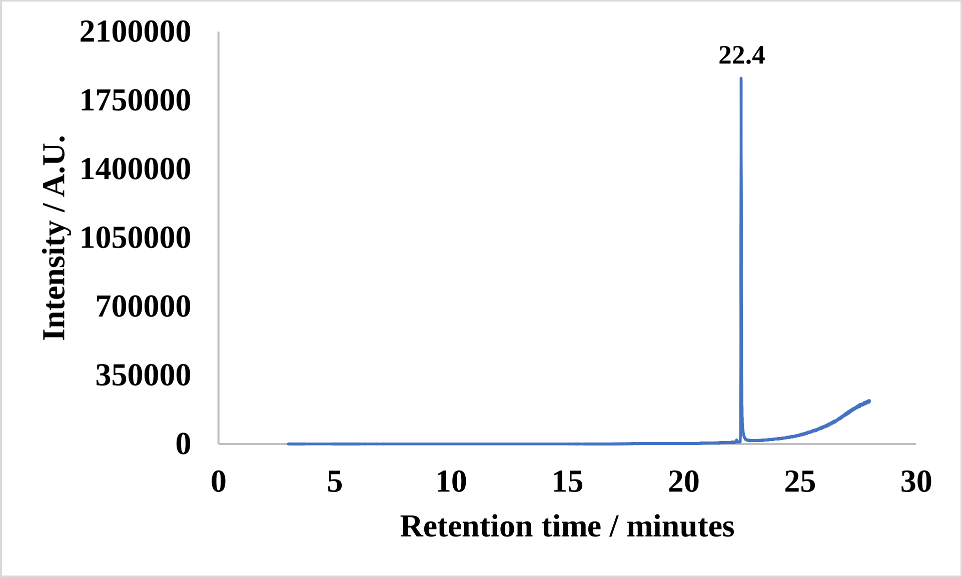

Fig. S36 GC chromatograms of photocatalytic selective oxidation of β-O-4 alcohol (2-(2-methoxyphenoxy)-1-(4-methoxyphenyl)ethanol) product in acetonitrile.

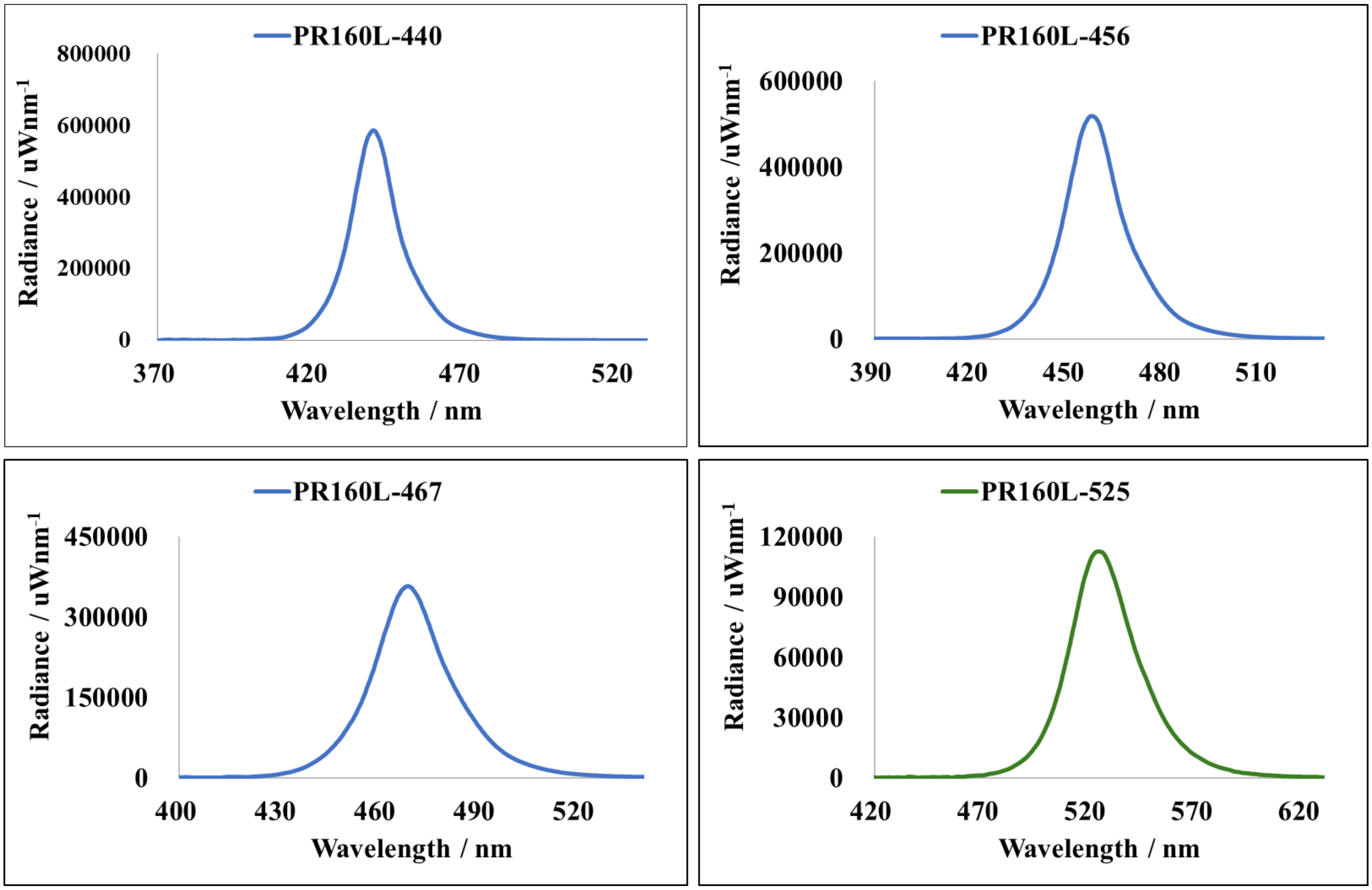

Fig. S37. Emission spectra of light sources (Kessil PR-160L)

2-(2-methoxyphenoxy)-1-(4-methoxyphenyl)ethanone

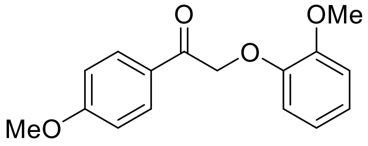

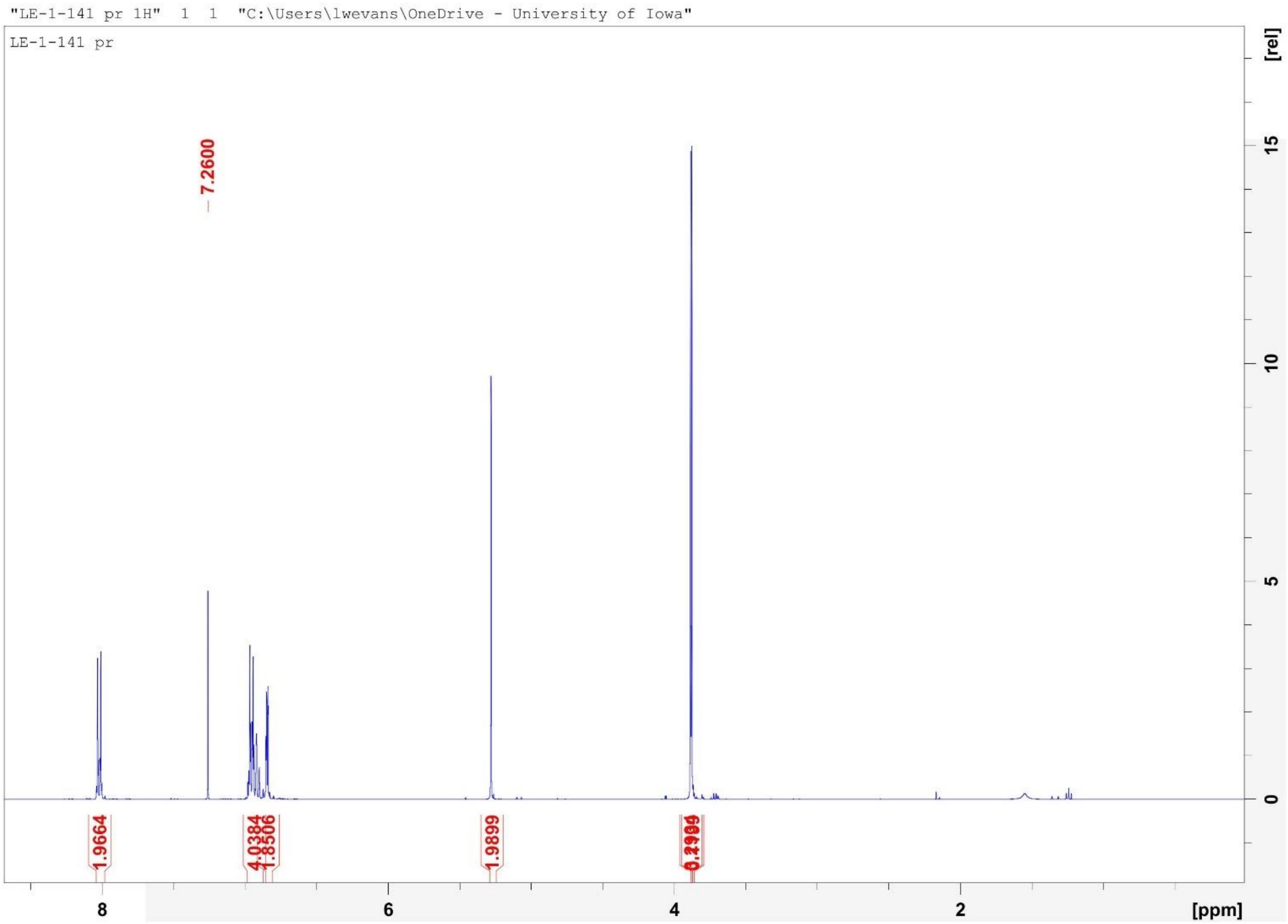

2-phenoxy-1-phenylethanone

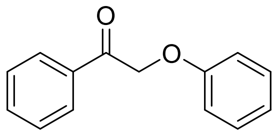

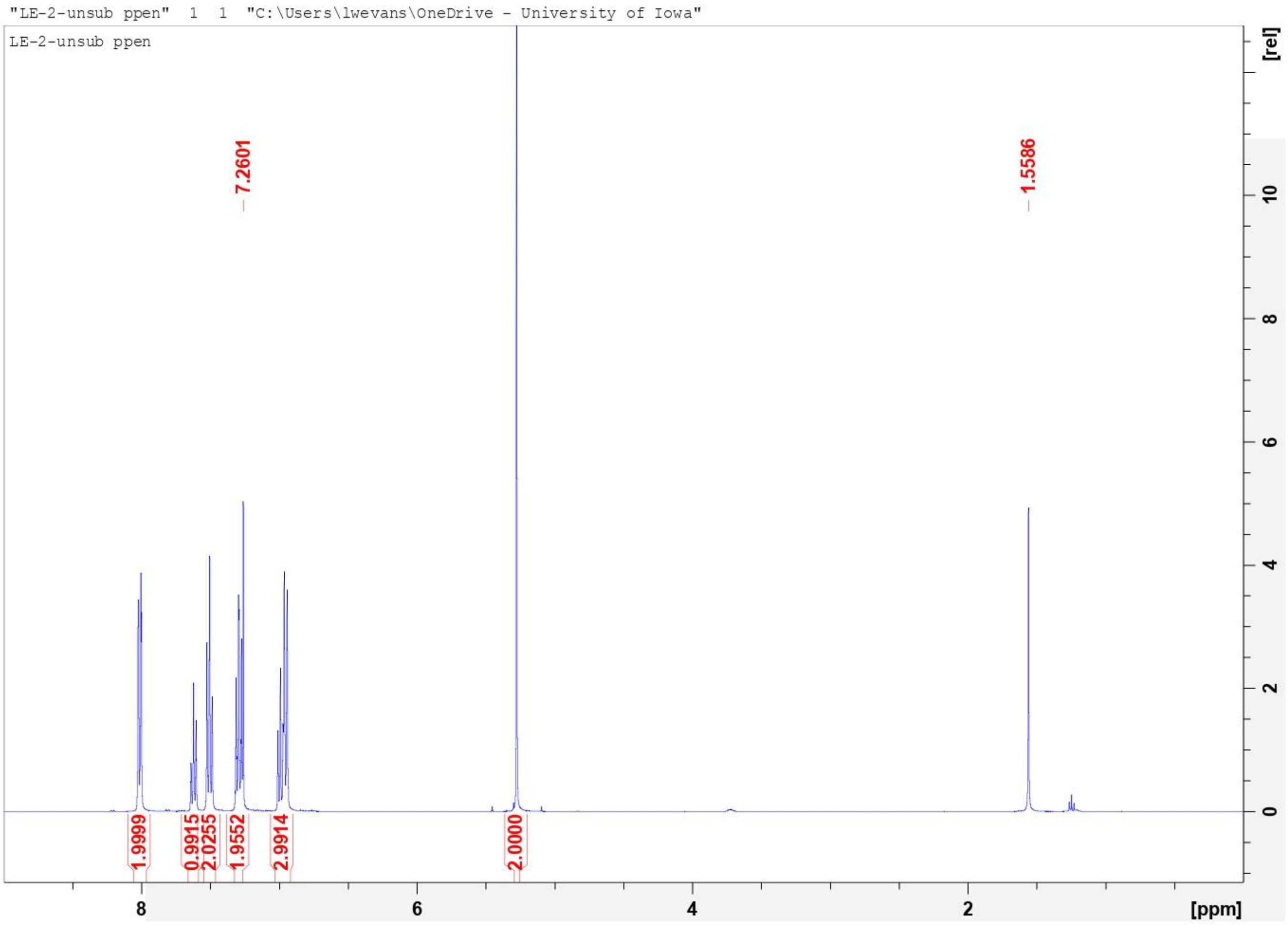

2-(2-methoxyphenoxy)-1-phenylethanone

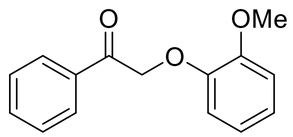

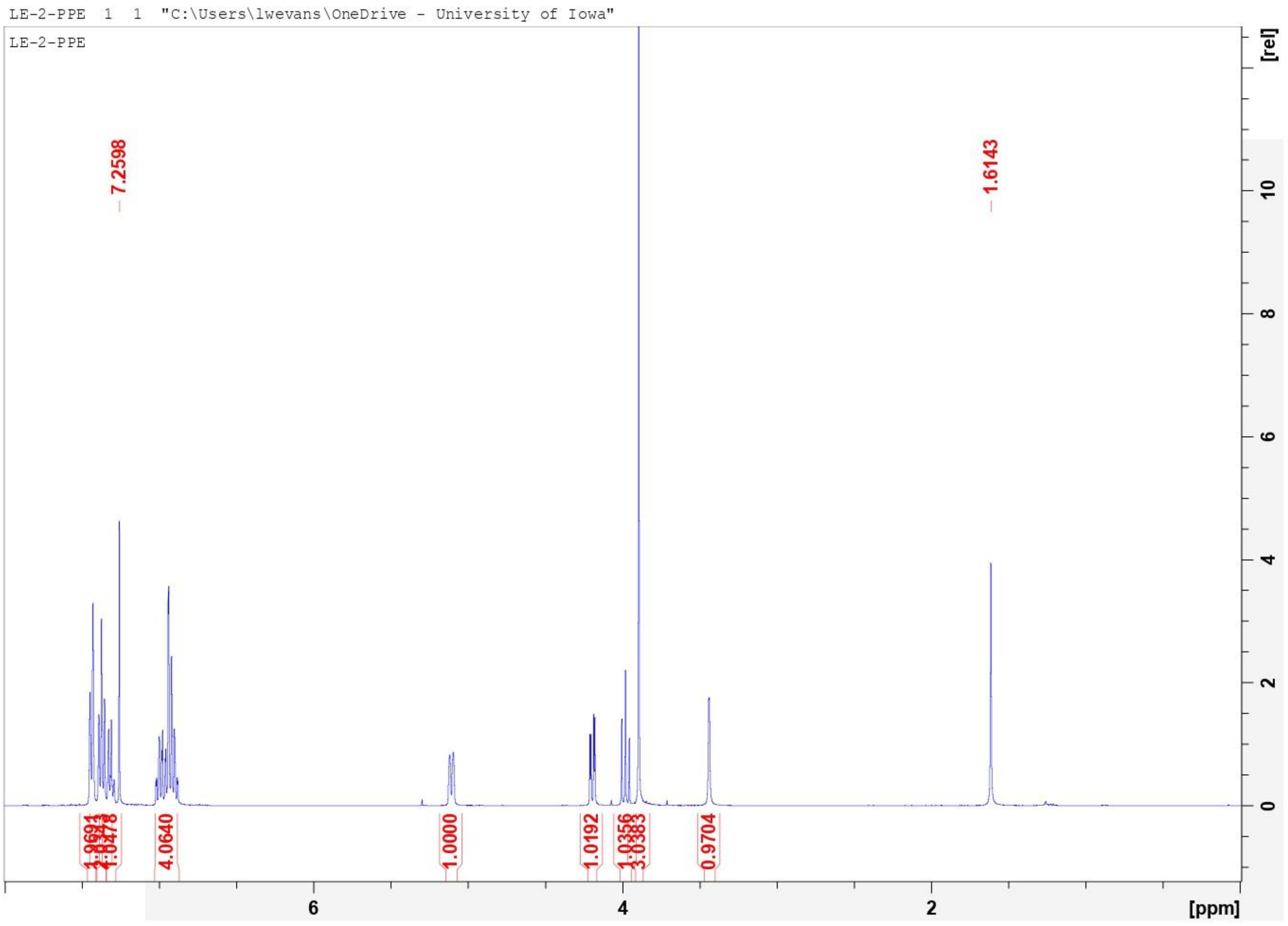

2-(2-methoxyphenoxy)-1-phenylethanol

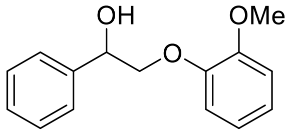

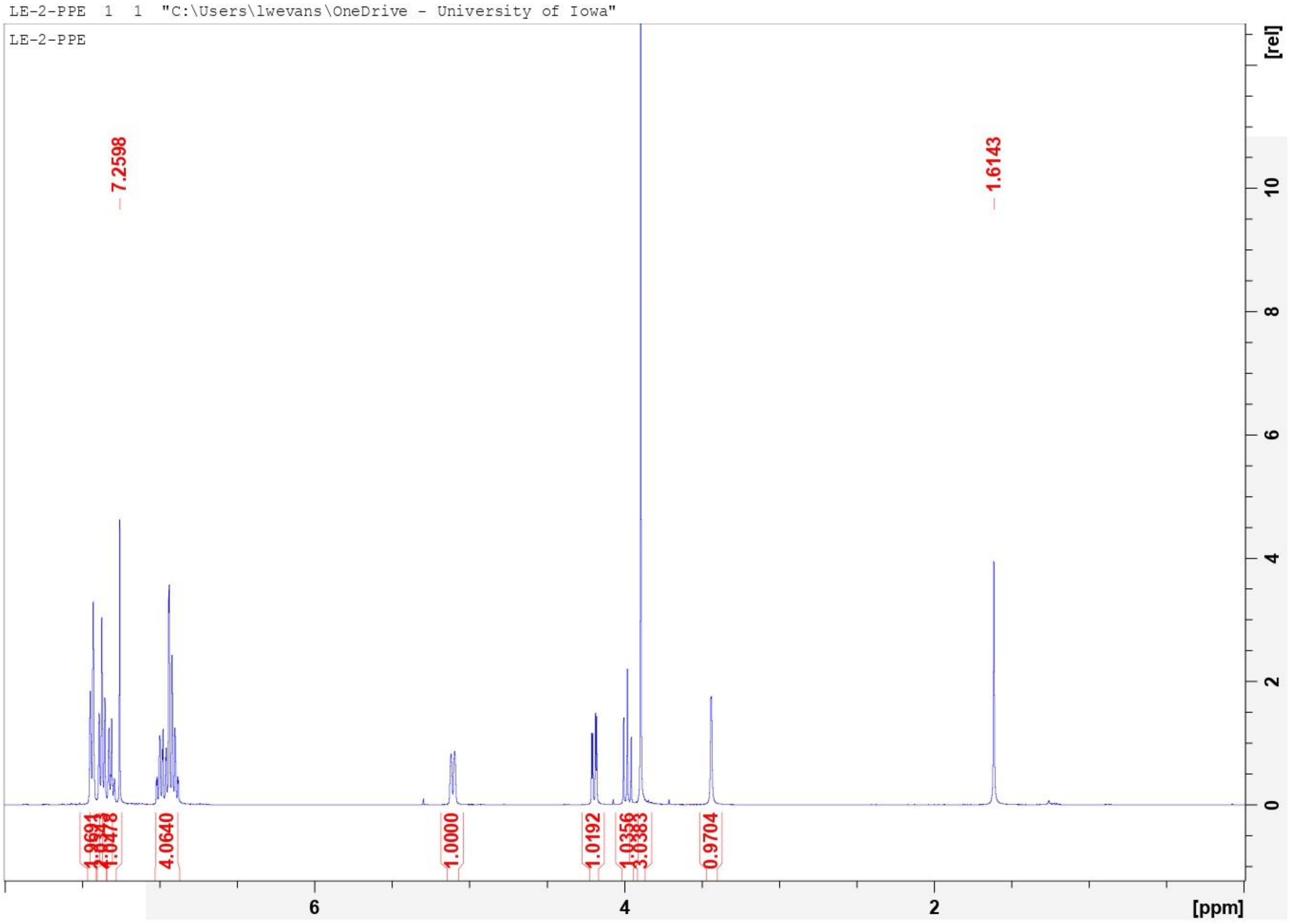

2-(2-Methoxyphenoxy)-1-(4-methoxyphenyl)ethanol

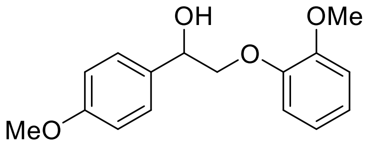

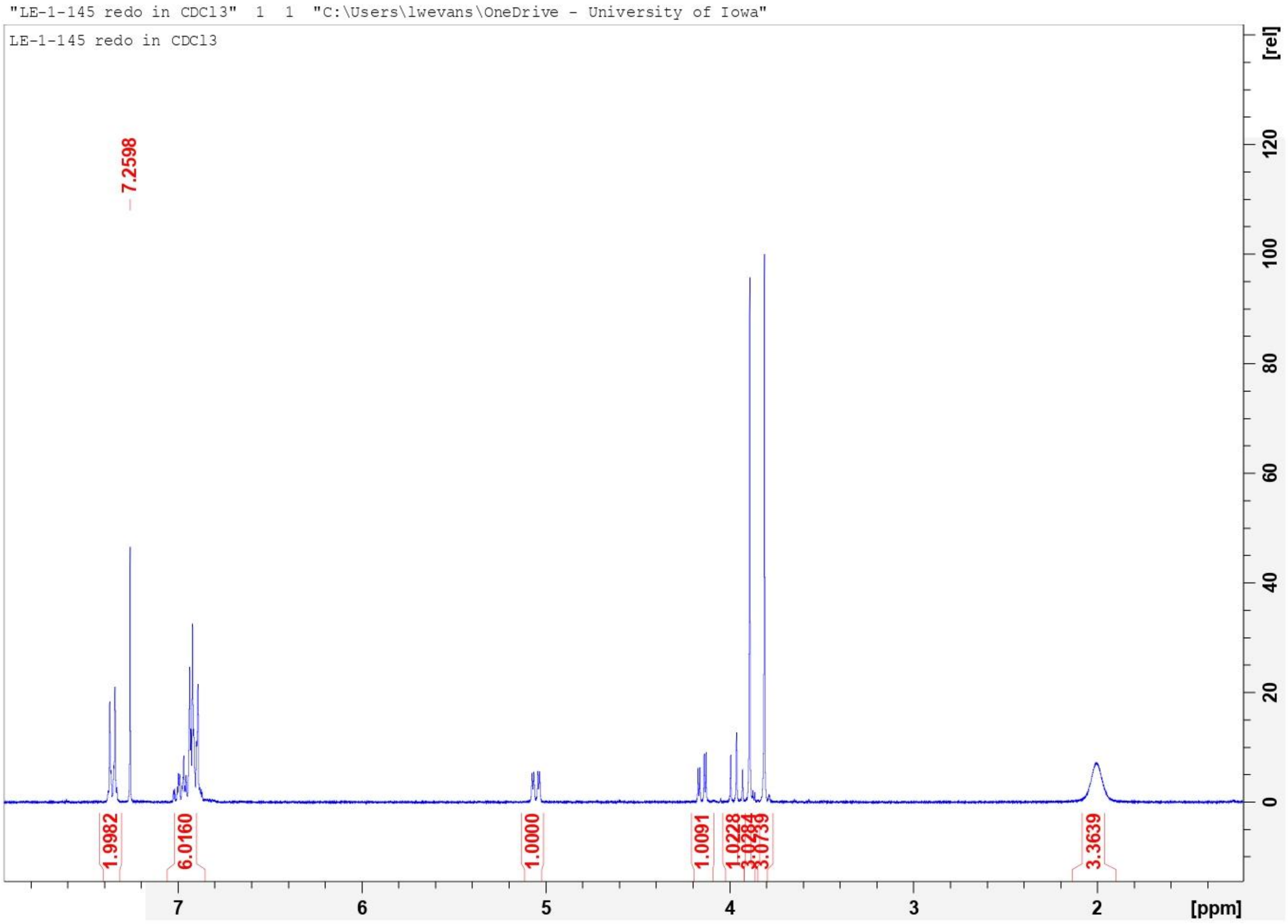

References:

1 J. M. Nichols, L. M. Bishop, R. G. Bergman and J. A. Ellman, *J. Am. Chem. Soc.*, 2010, **132**, 12554–12555.

2 M. D. Kärkäs, I. Bosque, B. S. Matsuura and C. R. J. Stephenson, *Org. Lett.*, 2016, **18**, 5166–5169.

3 M. J. Enright, K. Gilbert-Bass, H. Sarsito and B. M. Cossairt, *Chem. Mater.*, 2019, **31**, 2677–2682.

4 S. P. Pitre, C. D. McTiernan, W. Vine, R. DiPucchio, M. Grenier and J. C. Scaiano, *Sci. Rep.*, 2015, **5**, 16397.

5 A. Khan, M. Goepel, A. Kubas, D. Łomot, W. Lisowski, D. Lisovytskiy, A. Nowicka, J. C. Colmenares and R. Gläser, *ChemSusChem*, 2021, **14**, 1351–1362.

6 Y. T. He, D. Kang, I. Kim and S. Hong, *Green Chem.*, 2018, **20**, 5209–5214.

7 N. Luo, M. Wang, H. Li, J. Zhang, T. Hou, H. Chen, X. Zhang, J. Lu, and F. Wang. *ACS Catal.*, 2017, **7**, 4571-4580
